# Analysis of Mutations in *Neurospora crassa* ERMES Components Reveals Specific Functions Related to β-Barrel Protein Assembly and Maintenance of Mitochondrial Morphology

**DOI:** 10.1371/journal.pone.0071837

**Published:** 2013-08-05

**Authors:** Jeremy G. Wideman, Sebastian W. K. Lackey, Martin A. Srayko, Kacie A. Norton, Frank E. Nargang

**Affiliations:** Department of Biological Sciences, University of Alberta, Edmonton, Alberta, Canada; Arizona State University, United States of America

## Abstract

The endoplasmic reticulum mitochondria encounter structure (ERMES) tethers the ER to mitochondria and contains four structural components: Mmm1, Mdm12, Mdm10, and Mmm2 (Mdm34). The Gem1 protein may play a role in regulating ERMES function. *Saccharomyces cerevisiae* and *Neurospora crassa* strains lacking any of Mmm1, Mdm12, or Mdm10 are known to show a variety of phenotypic defects including altered mitochondrial morphology and defects in the assembly of β-barrel proteins into the mitochondrial outer membrane. Here we examine ERMES complex components in *N. crassa* and show that Mmm1 is an ER membrane protein containing a Cys residue near its N-terminus that is conserved in the class Sordariomycetes. The residue occurs in the ER-lumen domain of the protein and is involved in the formation of disulphide bonds that give rise to Mmm1 dimers. Dimer formation is required for efficient assembly of Tom40 into the TOM complex. However, no effects are seen on porin assembly or mitochondrial morphology. This demonstrates a specificity of function and suggests a direct role for Mmm1 in Tom40 assembly. Mutation of a highly conserved region in the cytosolic domain of Mmm1 results in moderate defects in Tom40 and porin assembly, as well as a slight morphological phenotype. Previous reports have not examined the role of Mmm2 with respect to mitochondrial protein import and assembly. Here we show that absence of Mmm2 affects assembly of β-barrel proteins and that lack of any ERMES structural component results in defects in Tom22 assembly. Loss of *N. crassa* Gem1 has no effect on the assembly of these proteins but does affect mitochondrial morphology.

## Introduction

Regions of close apposition between the endoplasmic reticulum (ER) and mitochondria have been observed for several years and are thought to be required for lipid and calcium exchange between the two organelles [1], [2], [3], [4], [5], [6]. Recently, interactions between proteins in the ER membrane and the outer mitochondrial membrane that could act as tethers between the two organelles have been described. In mammals, two sets of interactions have been reported. The first involves an interaction between the ER calcium channel IP_3_R (inositol 1,4,5-triphosphate receptor) and the mitochondrial VDAC (voltage dependent anion channel) protein that also involves a chaperone [7]. In the second, ER-localized Mfn2 tethers mitochondria to the ER by homotypic and heterotypic interactions with mitochondrially localized Mfn2 or Mfn1 [8]. In *Saccharomyces cerevisiae* the ER-mitochondria encounter structure (ERMES) has been shown to tether the two organelles [9], [10]. The ERMES is composed of four interacting structural proteins: the ER membrane protein, Mmm1; two mitochondrial outer membrane (MOM) proteins, Mmm2 (Mdm34) and Mdm10; and the cytosolic bridge protein, Mdm12. The Gem1 protein has also been reported to co-purify with the ERMES and it may play a role in regulating the size, organization, and function of the complex [11], [12]. However, a different study concluded that Gem1 is not involved in ERMES assembly or maintenance [10].

The genes encoding the four structural ERMES proteins of *S. cerevisiae* were originally identified in genetic screens for mutants with defects in mitochondrial distribution and morphology [13], [14], [15], [16], [17]. Localization studies and analysis of mutants have suggested that each of the proteins is involved in several cellular functions. Strains lacking any one of the four proteins were found to contain large spherical condensed mitochondria, exhibited defects in mitochondrial inheritance, showed loss of mtDNA, and had altered ratios of mitochondrial phospholipids [9], [13], [14], [15], [16], [17], [18], [19]. Mdm10, Mdm12, and Mmm1 were also required for mitochondrial motility mediated by attachment to the actin cytoskeleton [20], [21]. Fluorescence microscopy studies showed that Mdm10, Mdm12, and Mmm1 co-localized with mtDNA nucleoids [21], [22], [23]. Specific mutant alleles of MDM10 and MMM1 have also been found to increase the rate of mtDNA migration to the nucleus [18]. Unexpectedly, mutants lacking Mmm1, Mdm12, or Mdm10 were also shown to have defects in the assembly of β-barrel proteins into the MOM [24], [25], [26]. The process of β-barrel assembly is accomplished by the TOB complex (topogenesis of β-barrel proteins), which is also known as the SAM complex (sorting and assembly machinery). The Mdm10 protein has been shown to associate with the TOB/SAM complex [24], [25], [26], [27], [28] in addition to being a component of the ERMES. The outer membrane protein Tom22, which has one α-helical membrane spanning domain, has also been identified as a TOB complex substrate [29], [30] and defects in the assembly of this TOM complex protein have also been noted in *S. cerevisiae* strains lacking Mdm10 [25]. In other fungi, such as *Aspergillus nidulans*, *Podospora anserina*, and *Neurospora crassa*, alterations in mitochondrial morphology have been observed in mutants affecting the orthologues of the *S. cerevisiae* ERMES proteins [26], [31], [32], [33]. The β -barrel assembly defects and association of Mdm10 with the TOB complex have also been documented in *N. crassa* [26], [27].

The array of possible functions for ERMES components suggested by these studies has led to the problem of distinguishing primary effects from those which might be an indirect consequence of others. In this regard, it has been shown that in a strain containing a temperature-sensitive allele of *mmm1*, β-barrel assembly defects precede morphological alterations following a shift from the permissive to the restrictive temperature [24]. Similarly, mitochondria containing low levels of an Mdm10 mutant protein had Tom40 assembly defects, but normal levels of phospholipids and only minor changes in mitochondrial morphology [34]. Another study concluded that the primary function of ERMES was to maintain the link between ER and mitochondria [10]. In the absence of this link, mitochondrial morphology is altered which leads to secondary effects on mitochondrial inheritance. In the present study we have reasoned that if ERMES proteins are involved in different functions, then it should be possible to define domains in each protein responsible for at least some of those functions. We have examined the effects of mutations in two regions of the *N. crassa* Mmm1 protein with respect to mitochondrial morphology and TOB complex function. In addition, since there is currently controversy as to the role of the *S. cerevisiae* Gem1 protein in ERMES composition and function [10], [11], [12] we have also examined a *Δgem1 N. crassa* with respect to phenotypes characteristic of ERMES mutants. We extended our analysis to a strain lacking Mmm2 since, to our knowledge, mitochondrial protein import/assembly defects have never been examined for mutants lacking this ERMES complex member.

## Methods

### Strains and growth of *N. crassa*

Strains used in this study are listed in Table 1. Strains were grown at 30°C unless otherwise noted. General handling procedures for *N. crassa* [35] and growth tests using sorbose containing medium to force colonial growth were as described previously [36]. The deletion strains used in this study (*Δmmm1*, *Δmmm2*, and *Δgem1)* were developed by the *N. crassa* gene knockout project [37]. The target genes were replaced with a hygromycin resistance cassette. The strains were obtained from the fungal genetics stock center (FGSC). PCR analysis of genomic DNA was used to confirm the replacements in each strain.

**Table 1:** Strains used in this study.

Strain	Genotype	Origin and/or reference
NCN251	*A*	FGSC^1^ #2489
*Δmmm2*	*mmm2::hygR a*	FGSC #19795
*Δmmm1*	*mmm1::hygR a*	FGSC #21180
*mdm12*	*mdm12*	FGSC #9852 [70]
*Δmdm10*	*mdm10::hygR his-3 mtrR a*	[26]
*Δgem1*	*gem1::hygR a*	FGSC #19466
Mmm1-HA3	*mmm1::hygR a* Contains an ectopic copy of *mmm1* with a C-terminal 3xHA tag. Also Basta resistant	This study
Mmm1-HA5	As Mmm1-HA3 but expresses lower amounts of Mmm1-HA	This study
Mmm1-HA8	As Mmm1-HA3 but expresses lower amounts of Mmm1-HA	This study
Mmm1-Myc10	As Mmm1-HA3, but carries a C-terminal 3xMyc tag.	This study
CS-123	As Mmm1-HA3 but cysteine residues 5, 179 and 319 are mutated to serines	This study
C5S	As Mmm1-HA3 but cysteine residue 5 is mutated to serine	This study
C179S	As Mmm1-HA3 but cysteine residue 179 is mutated to serine	This study
C319S	As Mmm1-HA3 but cysteine residue 319 is mutated to serine	This study
A116-124	As Mmm1-HA3 but residues 116-124 are mutated to alanines	This study

1 Fungal Genetics Stock Center.

### Microscopy and measurement of mitochondrial diameter

*N. crassa* samples were prepared for visualization as previously described [26]. Imaging was done on an Olympus IX81 (60X, NA 1.42 oil objective) inverted microscope with a Yokogawa CSU-10 spinning disc confocal head modified with a condenser lens in the optical path (Quorum Technologies). Z-stacks (16 images, 0.2 µm spacing, ASI Nanodrive) were acquired for each hyphae. Digital images were obtained with a Hamamatsu Orca R2 camera, controlled by MetaMorph software (Molecular Devices). Post image processing and mitochondrial measurements were performed manually using Metamorph offline software (Molecular Devices). The length of individual mitochondria varied, therefore, to obtain width measurements of long mitochondria (>2 microns), multiple lines approximately 1 micron apart were drawn perpendicular to the long axis of the mitochondria. Optical planes used for width measurements of individual mitochondria were chosen based on maximum fluorescence intensity of MitoTracker. Measurements of mitochondria were obtained from at least four hyphae for each experiment. The mean hyphal widths of control and experimental populations were compared via a two-tailed Student’s t-test assuming unequal variances at p = 0.01.

### Creation of strains harboring mutant versions of Mmm1

The *mmm1* gene plus 500 bp of upstream and downstream sequence was cloned into *Asc*I sites in a modified Bluescript plasmid [38] containing basta resistance (pBasc). Using site directed PCR mutagenesis, a *Not*I site was inserted into the 3’ region of *mmm1* just before the stop codon. We designed another plasmid (FN-NotI-HA3) containing three repeats of the hemaglutinin (HA) epitope (YPYDVPDYA) flanked by *NotI* sites in a kanamycin resistance vector synthesized by Integrated DNA Technologies (IDT, Coralville, Iowa). One extra base pair was added on each side of the triple HA sequence to correct the frameshift caused by the insertion of two eight bp *Not*I sites. The additional nine bp (*Not*I site plus 1 bp) extensions on either side of the 3xHA sequence code for Ala-Ala-Ala and Gly-Gly-Arg, respectively. The triple HA tag was cut out using *Not*I and ligated into the *Not*I site that had been engineered in the *mmm1* gene. The resulting plasmid was called pMmm1-HA. An identical procedure was used to construct plasmid pMmm1-Myc except that plasmid FN-NotI-Myc3 was used as the source of a triple repeat of the Myc epitope (EQKLISEEDL).

Plasmid pMmm1-HA was subjected to site directed PCR mutagenesis to encode the desired mutant forms of Mmm1 described in Table 1. For C5S, C179S, and C319S Cys residues 5, 179, and 319 were changed to Ser, respectively. For CS-123, all three Cys residues were changed to Ser. For A116-124 residues 116-124 were each mutated to Ala. Plasmids containing mutations were confirmed by sequence analysis, linearized and used to transform Δ*mmm1*. The transformation mixtures were plated onto media containing basta. Strains were purified by one round of single colony isolation. Mutations were confirmed by sequence analysis of genomic DNA isolated from the transformants.

### Cellular fractionation experiments

Crude mitochondria were isolated as described previously [39]. In some experiments crude mitochondria were further purified in flotation sucrose gradients as described [40] except that the buffer used was SEMP (0.25 M sucrose, 1 mM EDTA, 10 mM MOPS, 1 mM phenylmethylsulfonyl fluoride (PMSF)) containing a cocktail of protease inhibitors (2μg/ml aprotinin, 1 µg/ml leupeptin and 1 µg/ml pepstatin A). The supernatant fraction from the initial crude mitochondrial preparation was further fractionated by subjecting 1 ml samples to ultracentrifugation at 130,000 x g at 4°C for 1.5 hr in a TLA55 rotor in a Beckman Optima™ MAX tabletop ultracentrifuge. The supernatant was collected as the cytosolic fraction. The pellet was termed the post mitochondrial pellet (PMP). Gel lanes were typically loaded with 30 µg of each fraction. Control marker proteins for the fractions were a TOM complex component for mitochondria, the endoplasmic reticulum protein KAR2 for PMP [41], and arginase for the cytosol [42], [43].

### Coimmunoprecipitation of differentially tagged Mmm1 proteins

Sterile distilled water was used to harvest fresh conidia from each of strain Mmm1-Myc10 and strain Mmm1-HA3. Both strains arose from transformation of *Δmmm1* and are of identical genetic background. Equal numbers of conidia (10^5^) from each strain were mixed and spotted at the center of 50 ml of agar solidified minimal Vogel’s medium in a 250 Erlenmeyer flask to generate a non-forced heterokaryon. Conidia from the culture were grown and a crude mitochondrial fraction was isolated from the mycelium as described [39], except that a protease inhibitor cocktail (described above) was also included in the isolation buffer. Mitochondria (1 mg) were resuspended in 250 µl buffer A (20 mM Tris-HCl pH 7.4, 0.1 mM EDTA, 50 mM NaCl, 2 mM PMSF) plus protease inhibitor cocktail, dissolved by addition of 250 µl of 2% digitonin, followed by gentle shaking for one hr at 4° C. The solution was then subjected to a clarifying spin at 13,000 rpm in a refrigerated microcentrifuge. The supernatant was collected and 10 µl of anti-Myc agarose beads (Thermo Scientific, Rockford, IL) were added followed by gentle mixing overnight at 4°C. The mixture was poured into a column, washed three times with 0.5 ml of Buffer B (20 mM Tris-HCl pH 7.4, 0.1 mM EDTA, 200 mM NaCl, 2 mM PMSF) plus protease inhibitor cocktail containing 0.2% digitonin, and eluted with three times 20 µl of 2X Laemli cracking buffer (0.125 M Tris-HCl, pH 6.8; 5% SDS; 5% β-mercaptoethanol; 5% sucrose). We found it impossible to elute Mmm1 constructs from the beads with elution buffers that did not contain β-mercaptoethanol. Control mitochondria from a non-tagged wild type strain (NCN251), the Mmm1-Myc10 homokaryotic strain, and the Mmm1-HA3 homokaryotic strain were processed in a similar fashion. The three elution fractions from each strain were pooled and subjected to SDS-PAGE. The gel was blotted to nitrocellulose and immunodecorated with anti-HA antibody.

### Phospholipid analysis by TLC (thin layer chromatography)

Mitochondrial phospholipids were extracted from isolated crude mitochondria (300 µg protein) resuspended in 100 µl water with 200 µL 1∶1 chloroform: methanol (v/v) using a procedure modified from Osman *et al*. [19]. Briefly, samples were vortexed for 30 sec and then shaken for 30 min. Samples were then centrifuged at 13, 000 x g on a table top centrifuge for 1 min to separate aqueous and organic phases. The aqueous phase was discarded and the organic phase was allowed to dry in a fume hood. The lipid pellet was then dissolved in 30 µl 2∶1 chloroform methanol (v/v) and subjected to TLC as previously described [44]. Briefly, samples were spotted onto TLC plates (5729-6 Merck KGaA, Darmstadt, Germany) that were prewashed in 1∶1 chloroform methanol (v/v) and developed in chloroform: water: ethanol: triethylamine (30∶7∶35∶35) until the liquid front neared the top of the plate (approximately 1.5 hr at room temperature). The plate was then allowed to dry in a fume hood and the developing step was repeated to increase resolution. Plates were dried a second time and sprayed with molybdenum blue spray reagent (M1942-100ML, Sigma). Phospholipid standards (P3556-25MG, P7943-5MG, C0563-10MG, Sigma) were dissolved in 1∶1 chloroform methanol and run beside experimental lanes to allow identification.

### General Procedures

Blue native gel electrophoresis (BNGE) [45], [46], Western blotting [47], import and assembly of proteins into isolated mitochondria that were not sucrose gradient purified (crude mitochondria) [48], alkaline extraction [26], [27], isolation of outer membrane vesicles [49], and transformation of *N. crassa* [36] were performed as described previously. Mitochondrial proteins were analyzed by SDS-PAGE as previously described [50]. However, where indicated, proteins were prepared for SDS-PAGE by dissolving in cracking buffer (0.06 M Tris-HCl, pH 6.8; 2.5% SDS; 5% sucrose) with (reducing) or without (non-reducing) 5% β-mercaptoethanol. In some cases irrelevant lanes were electronically removed from gel blots or autoradiograms.

## Results

### Mmm1 fractionates with both purified mitochondria and the ER but not with purified OMV

The Mmm1 protein was originally characterized as a MOM protein in both *S. cerevisiae* and *N. crassa* [16], [17], [22], [24], [33], [51]. However, more recent studies in *S. cerevisiae* have concluded that the protein resides in the ER membrane and associates with mitochondrial outer membrane proteins to form the ERMES [9], [11], [12]. We wished to investigate the location of Mmm1 in *N. crassa* by cell fractionation. Since no antibody to *N. crassa* Mmm1 is available, we constructed a plasmid containing the *N. crassa mmm1* gene with a C-terminal triple hemaglutinin (HA) epitope-tag expressed from the endogenous *mmm1* promoter. This construct was transformed into a *Δmmm1* strain and several transformants were examined on Western blots for the presence of Mmm1-HA. Although there was variation in the level of expression of the protein among the different transformants most expressed similar levels of the protein (Figure S1A). One such strain, Mmm1-HA3, was chosen for further work. The defects in mitochondrial morphology, growth rate, and β-barrel assembly that are seen in the *Δmmm1* strain [26] are all rescued in Mmm1-HA3 (Figures S1 B, C, D and S2).

**Figure 1:**
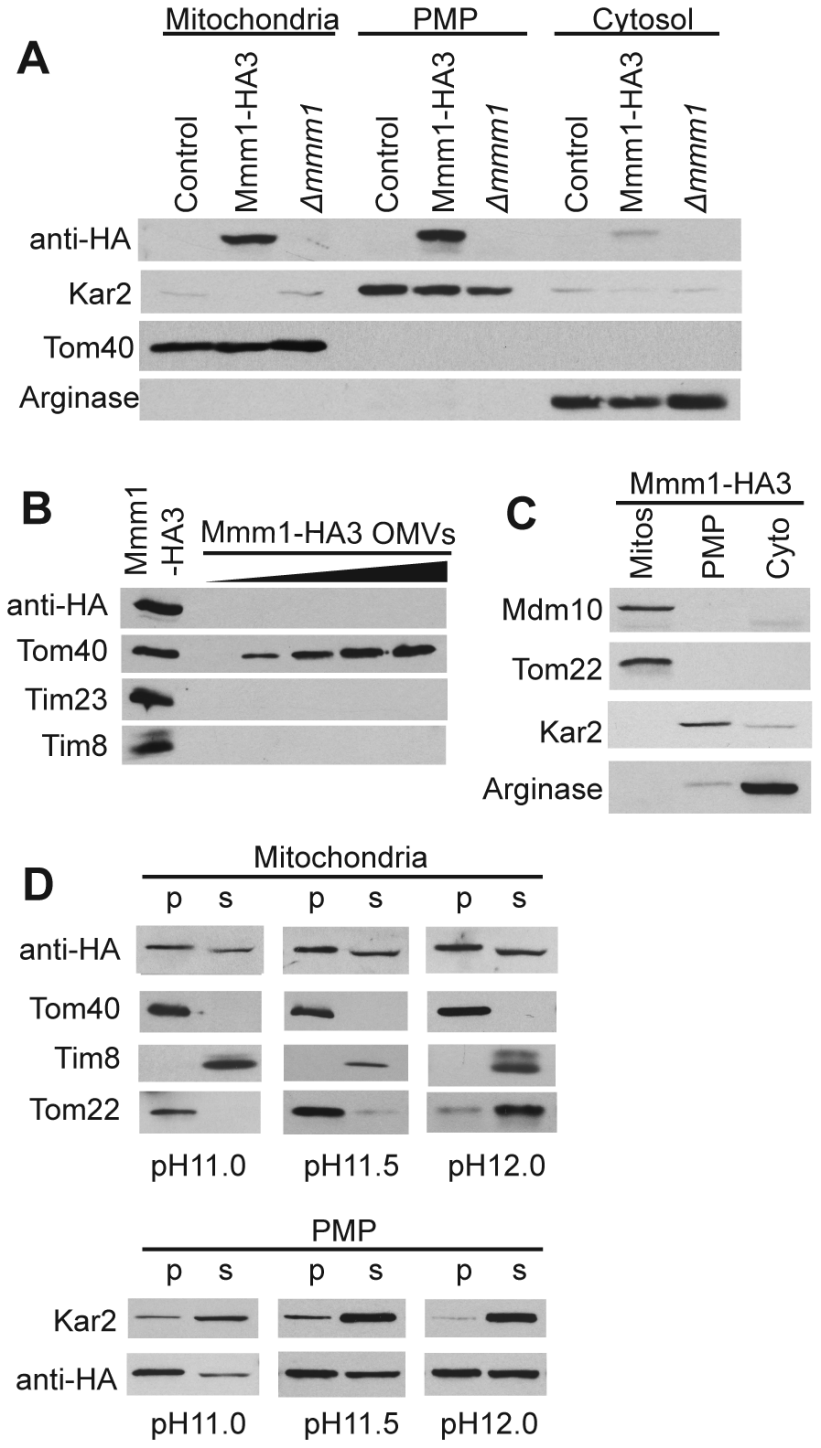
Subcellular localization of HA-tagged Mmm1. A. Cell fractionation by differential and sucrose flotation gradient centrifugation was performed on the indicated strains. Flotation gradient purified mitochondrial, post mitochondrial pellet (PMP) and cytosolic fractions (30 µg) were subjected to SDS-PAGE followed by Western blot analysis using antibodies to the indicated proteins. Kar2, ER marker; Tom40, mitochondrial marker; Arginase, cytosolic marker. B. Outer membrane vesicles (OMVs) were isolated from cells expressing Mmm1-HA protein, subjected to SDS-PAGE and analyzed by Western blot for the presence or absence of the indicated proteins. The leftmost lane contains whole mitochondria C. Cell fractionation and detection of indicated proteins. As in panel A but Tom22 is the mitochondrial marker. D. Flotation gradient purified mitochondria and PMP isolated from the Mmm1-HA strain were treated with 0.1 M sodium carbonate at pH 11.0, 11.5 or 12.0. Membrane sheets were pelleted by ultracentrifugation. Proteins in the supernatant were precipitated with trichloroacetic acid. Pellet (pel) and supernatant (sup) fractions were then subjected to SDS-PAGE, transferred to nitrocellulose and analyzed by Western blot using antibodies to the indicated proteins.

Isolation of subcellular fractions from the tagged strain revealed that Mmm1-HA fractionated with both flotation gradient purified mitochondria and the post-mitochondrial pellet (PMP), which contains the ER as shown by the presence of the Kar2 marker protein [41] in this fraction (Figure 1A). These data are consistent with the notion that Mmm1 is an ER-membrane protein that interacts with MOM proteins but do not exclude the possibility that it is a MOM protein that interacts with the ER. To distinguish between these two alternatives we examined mitochondrial OMV for the presence of Mmm1-HA. If Mmm1 was a bona fide MOM protein it would fractionate with OMV. On the other hand, if Mmm1 was an ER protein, then it seemed likely that its association with gradient purified mitochondria in our fractionation experiments would be due to small patches of ER membrane that stay associated with mitochondria at ERMES attachment points during isolation and gradient purification. However, since OMV are isolated by shearing the MOM from mitochondria followed by purification based on density in sucrose gradients [49], membrane fragments containing both MOM and ER would be expected to separate from fragments made up solely of MOM. Western blot analysis of OMV prepared from Mmm1-HA3 revealed the presence of the MOM protein Tom40, whereas Mmm1-HA, the inner mitochondrial membrane protein Tim23, and the intermembrane space protein Tim8 could not be detected (Figure 1B). This result is consistent with the observation that Mmm1 was undetectable in *N.crassa* OMVs by mass spectrometry [52]. Conversely, we have previously shown that Mdm10, another ERMES component, does fractionate with mitochondrial OMVs [26]. Here we have also shown that Mdm10 only fractionates with gradient purified mitochondria and not the PMP (Figure 1C). Taken together, these data suggest that Mmm1 is an ER protein. During subcellular fractionation, fragments of ER membrane appear to stay attached to mitochondria at ERMES junctions. They also suggest that there are two populations of Mmm1. One population is tightly associated with mitochondria while the other is not, and fractionates with the PMP.

We also performed alkali extractions to determine if Mmm1 behaves differently with respect to membrane integration in the gradient purified mitochondria versus the PMP fractions. Mmm1 was approximately 50% extractable at pH 12.0 in both fractions (Figure 1D). Since the known membrane spanning protein Tom22 is almost completely extracted from mitochondria at this pH it appears that Mmm1 is strongly anchored to the ER membrane. From these data and previous data from *S. cerevisiae* [9], [11], [12] we conclude that while a population of Mmm1 molecules fractionates with mitochondria, Mmm1 is actually an ER membrane protein.

### Mmm1 structure and function

**Figure 2:**
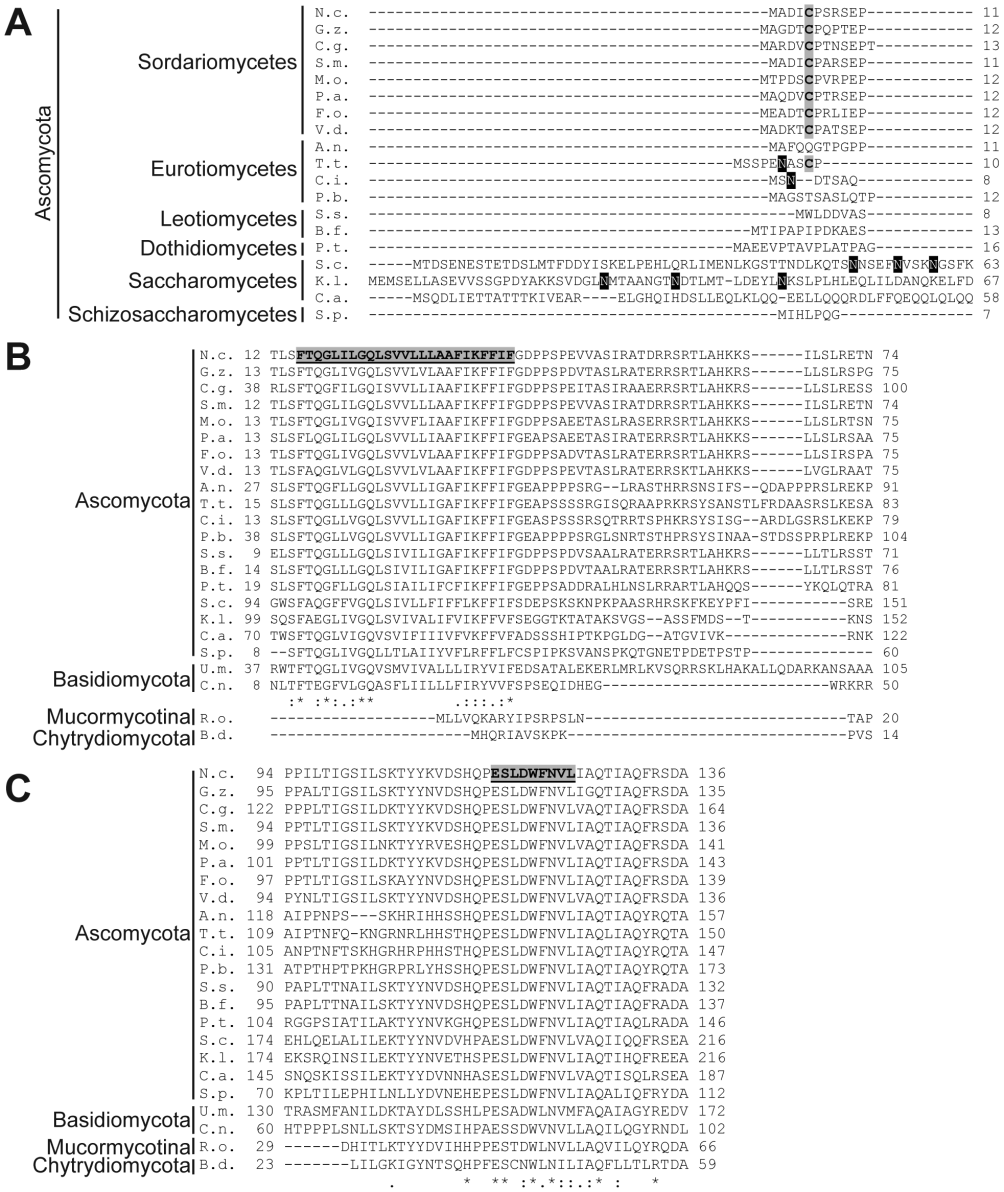
Alignments of fungal Mmm1 proteins. A. Alignment of Mmm1 N-terminal regions from several Ascomycetes. Known *S. cerevisiae* (N50, N55 and N59) [12] and potential N-glycosylation sites in other species are highlighted in black. Cys residue conserved in Sordariomycetes is shaded in grey. B. Predicted transmembrane domain of Mmm1is present in most fungi, but absent in Mucormycotina and Chytridiomycota. The predicted *N. crassa* transmembrane domain is highlighted in grey. Identity/similarity symbols are for the alignment of the Ascomycota and Basidiomycota only. * indicates conserved residues, : indicates conservation of groups with strongly similar properties (score of >0.5 in the Gonnet PAM 250 matrix),. indicates conservation of groups with weakly similar properties (score of <0.5 in the Gonnet PAM 250 matrix). C. Alignment of the highly conserved region of Mmm1 chosen for mutation analysis. The nine amino acid region that was chosen for mutation is highlighted in the *N. crassa* protein (residues 116-124). Symbols (as in panel B) are for the alignment of all proteins. Abbreviations: N.c., *Neurospora crassa*; G.z., *Gibberella zeae*; C.g., *Chaetomium globosum*; S.m., *Sordaria macrospora*; M.o., *Magnaporthe oryzae*; P.a., *Podospora anserina*; F.o. *Fusarium oxysporum*; V.d., *Verticillium dahliae*; A.n., *Aspergillus nidulans*; T.t.,*Trichophyton tonsurans*; C.i., *Coccidioides immitis*; P.b., *Paracoccidioides brasiliensis*; S.s., *Sclerotinia sclerotiorum*; B.f., *Botryotinia fuckeliana*; P.t., *Pyrenophora teres*; S.c., *Saccharomyces cerevisiae*; K.l., *Kluyveromyces lactis*; C.a., *Candida albicans*; S.p., *Schizosaccharomyces pombe*; U.m., *Ustilago maydis*; C.n., *Cryptocuccus neoformans*; R.o., *Rhizopus oryzae*; B.d., *Batrachochytrium dendrobatidis*.

Mmm1 proteins are quite well conserved among different fungi. Most species we examined contain a small N-terminal domain (Figure 2A) followed by a predicted membrane spanning domain (Figure 2B), and then a large C-terminal domain (Figure 2C, Figure S3). The latter has been shown to exist in the cytosol in *S. cerevisiae* and *N. crassa* [16], [33] and is known to be essential for function [12], [16], [51]. The cytosolic domain contains a synaptotagmin-like-mitochondrial-lipid binding protein (SMP) domain, which may be involved in lipid transfer between ER and mitochondria [53], and has been shown to be essential for protein targeting to organelle contact sites [54]. The N-terminal region of *S. cerevisiae* Mmm1 that precedes the membrane spanning domain is located in the ER lumen and is known to be N-glycosylated [9], [12] (Figure 2A). However, in many species the ER-lumen domain is much smaller than in *S. cerevisiae* and lacks any predicted N-glycosylation sites (Figure 2A). Surprisingly, species from the subphylum Mucormycotina and the phylum Chytridiomycota appear to lack a membrane spanning domain altogether (Figure 2B) and therefore must also lack the ER-lumen domain. If the Mmm1 protein in these groups serves the same function as the *N. crassa* and *S. cerevisiae* homologs, it may be as a strongly associated peripheral membrane protein of the ER.

**Figure 3:**
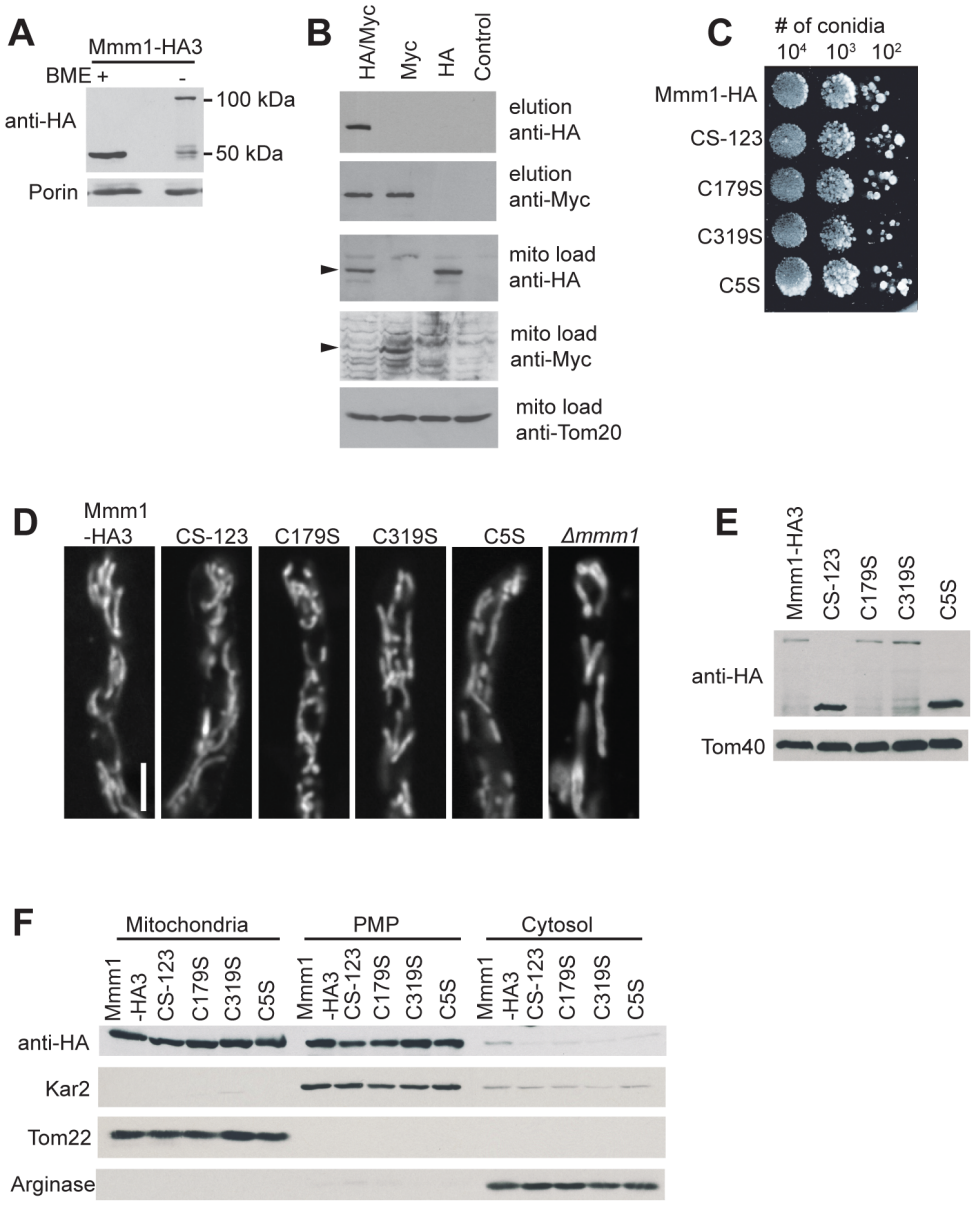
Characterization of *N. crassa* Cys to Ser Mmm1 mutants. A. The Mmm1-HA protein engages in disulphide bonding. Mitochondria (30 µg) were treated with cracking buffer that either did (+BME) or did not (-BME) contain β-mercaptoethanol. Samples were subjected to SDS-PAGE, transferred to nitrocellulose and analyzed by Western blotting for the indicated proteins. B. Coimmunoprecipitation of two tagged forms of Mmm1 from a heterokaryon. An unforced heterokaryon consisting of strains Mmm1-HA3 and Mmm1-Myc10 (HA/Myc) was constructed as described in the Methods. Mitochondria isolated from the heterokaryon were dissolved and treated with anti-Myc agarose beads. Elutions from the beads, or total mitochondrial proteins (mito load, to monitor the input level of proteins), were electrophoresed, blotted, and immunodecorated with the antibodies indicated on the right. Controls were an untagged wild type NCN251 strain (control), the homokaryotic Mmm1-HA3 strain (HA), and the homokaryotic Mmm1-Myc10 strain (Myc). Arrowheads on the left indicate the relevant bands in panels containing non-specific background bands. C. Tenfold dilutions of conidiaspores from strains expressing control (Mmm1-HA) and mutant HA-tagged versions of Mmm1 were spotted on plates containing Vogel’s sorbose medium. The plates were incubated at 30°C for 48 h and then photographed. D. Strains expressing control (Mmm1-HA) and mutant HA-tagged versions of Mmm1 were grown on solid Vogel’s media, stained with MitoTracker Green FM and examined by confocal fluorescence microscopy. Mitochondria in the *Δmmm1* strain are shown for comparison. Bar represents 10 µm. E. Western blot analysis of Mmm1 Cys mutant crude mitochondria. As in Figure 3A, but mitochondria were only analyzed by non-reducing SDS-PAGE. F. Cell fractionation of the indicated strains as described in Figure 1A except that Tom22 was the mitochondrial marker.

Further examination of the Mmm1 alignment revealed that two Cys residues were conserved in many Ascomycetes (position 179 and 319 of the *N. crassa* protein) while a third, at position 5 of the *N. crassa* protein, was conserved in the class Sordariomycetes (Figure 2A and Figure S3). Since the domain containing the C5 residue is predicted to be located in the ER lumen, it was possible that it might be involved in disulphide bond formation. When isolated mitochondria (Figure 3A) or PMP (data not shown) were analyzed on Western blots following non-reducing SDS-PAGE, a band was detected at approximately 100 kDa, twice the molecular weight of Mmm1-HA. A small amount of material was still seen at 50 kDa, but the majority of the Mmm1 protein appears to be in a disulphide bonded form, most likely a dimer. To verify that the 100 kDa form was an Mmm1 dimer, we performed a coimmunoprecipitation experiment using a heterokaryotic strain formed between Mmm1-HA3 and a strain constructed to express only a Myc-tagged version of the protein, Mmm1-Myc10. Mitochondria isolated from the heterokaryon were dissolved in 1% digitonin and incubated with anti-Myc antibody bound to agarose beads. As shown in Figure 3B, HA-tagged Mmm1 protein was coimmunoprecipitated with the Myc-tagged protein.

### Loss of disulphide bond formation in the ER-lumen domain of Mmm1 affects Tom40 assembly

To determine if Mmm1 disulphide bonding is functionally relevant in *N. crassa* we used the HA-tagged wild type gene to construct mutant versions of the protein in which each of the three Cys residues was changed individually to Ser (C5S, C179S and C319S) as well as a version in which all three Cys were changed to Ser (CS-123). The *Δmmm1* strain was transformed with plasmids encoding these mutant versions of the protein. Strains expressing any of the Cys mutation proteins grew at wild type rates (Figure 3C) and had normal mitochondrial morphology (Figure 3D, Figure S2). Mitochondria were isolated from transformants and analysed by non-reducing SDS-PAGE. As expected, the Cys responsible for disulphide bond formation of Mmm1 was the one predicted to occur in the oxidizing environment of the ER, at position 5 of the *N. crassa* protein (Figure 3E). Fractionation experiments showed that none of the Cys mutations result in mislocalization of Mmm1 (Figure 3F).

**Figure 4:**
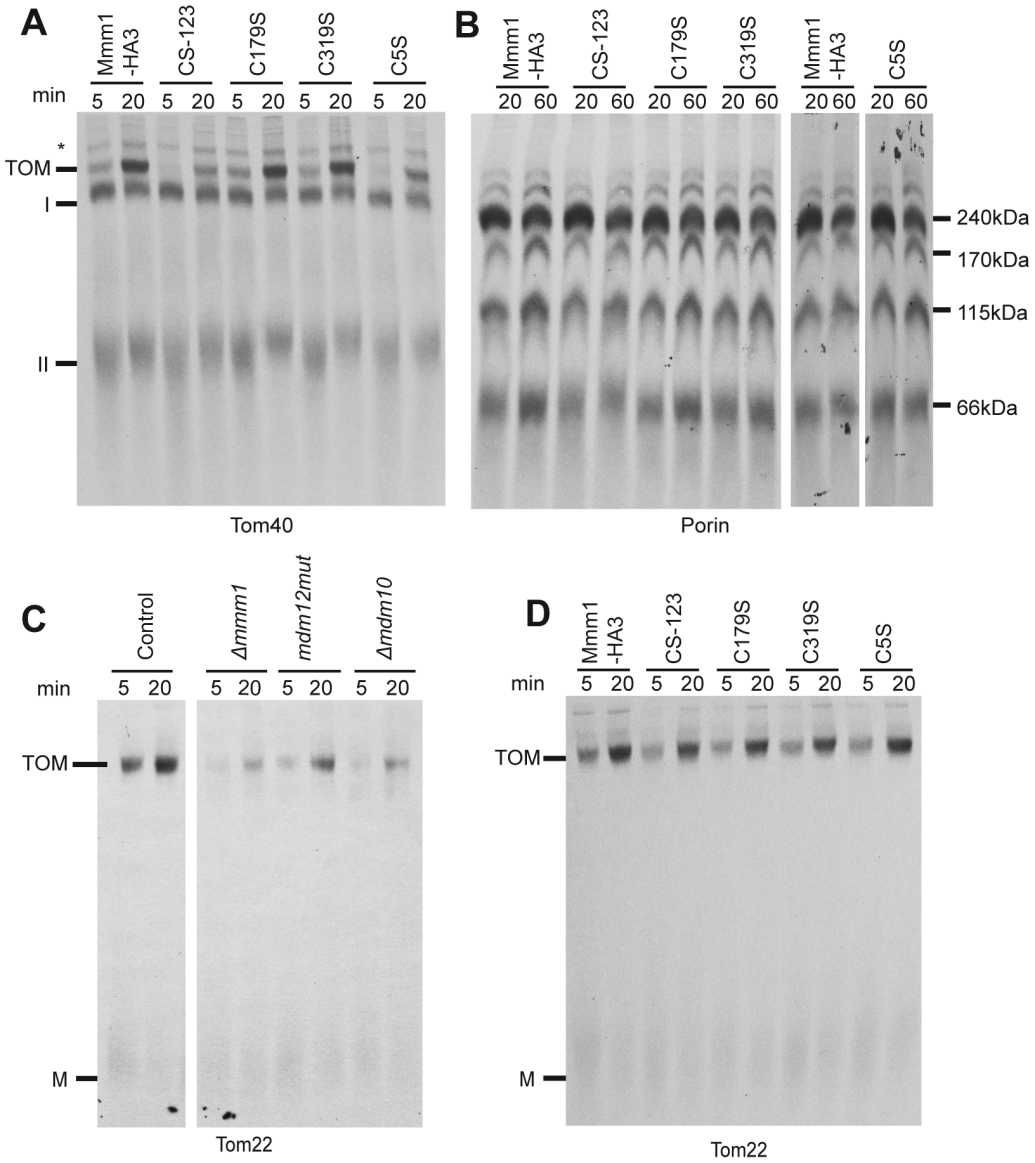
Assembly of mitochondrial outer membrane proteins into Mmm1 Cys to Ser mutants. A. Import and assembly of Tom40 was assayed by incubating radiolabeled Tom40 precursor with isolated mitochondria for the indicated times. After import, the mitochondria were reisolated and dissolved in 1% digitonin. The samples were subjected to BNGE, transferred to PVDF membrane and analyzed by autoradiography. The positions of the mature TOM complex, intermediates I and II, and an unknown band (*) are indicated. The time (min) of each import reaction is indicated above the lanes. B. As in A, except radiolabelled porin precursor was imported. The molecular masses of the major complexes containing radiolabeled porin are indicated. C. As in A, but radiolabeled Tom22 precursor was imported into isolated mitochondria from the indicated strains. Mature TOM complex and monomeric Tom22 (M) are indicated. D. Assembly of Tom22 into mitochondria from the indicated strains as in panel C.

We next examined mitochondria isolated from the Cys mutants for their ability to assemble Tom40 and porin into the MOM, since these β-barrels are known to have reduced assembly in Mmm1 mutants ([26], Figure S1). These proteins are inserted into the membrane by the TOB complex. The assembly of Tom40 progresses through known stages that are observable when analyzed by BNGE [55], [56], [57]. Radiolabeled Tom40 precursor can first be seen following its import through the MOM when it interacts with the TOB complex as intermediate I at 250 kDa. From there it is released into the outer membrane where it interacts with endogenous molecules of Tom5 and Tom6 to form a 100 kDa complex termed intermediate II. Intermediate II is then assembled into the mature 400 kDa TOM complex. We found that the inability of Mmm1 to form disulphide bonds in the C5S and CS-123 mutants caused a moderate defect in the incorporation of Tom40 into the final TOM complex (Figure 4A).

Several complexes are also detected when porin import/assembly is assessed by BNGE. Four prominent bands and other minor bands are seen, though the nature of the different forms is not well understood. Assembly of porin (Figure 4B) did not appear to be altered in the C5S or CS-123 mutants. Since Tom22 is also a substrate of the TOB complex [25], [29], [30], [58] we wished to assess its assembly in the C5S mutant. However, *N. crassa* ERMES mutants had not been previously tested for Tom22 assembly, so we first examined mitochondria from the *Δmmm1* mutant as well as the previously described *mdm12* mutant and *Δmdm10* strain. Mitochondria from all three mutant strains exhibited reduced ability to assemble Tom22 into the TOM complex (Figure 4C). On the other hand, mitochondria from all the Cys mutants of Mmm1 assembled Tom22 as effectively as the control mitochondria (Figure 4D). Thus, the ability of Mmm1 to form disulphide bonds differentially affects aspects of the Δ*mmm1* phenotype.

It might be argued that the amino acid substitutions in the C5S and CS-123 mutants leads to a deficiency in TOM or TOB complex components which, in turn, would result in the Tom40 assembly defects. However, we consider this extremely unlikely since the *Δmmm1* strain contains normal levels of these components [26]. Furthermore, we have shown that the TOM complex (Figure S4A) and the intermembrane space chaperone proteins Tim8 and Tim13 (Figure S4B,C) are present at normal levels in the C5S mutant.

The above data demonstrate that the ER-lumen domain of *N. crassa* Mmm1 has functional importance. However, it is not clear that this would extend to other fungal species because of the considerable variation within the N-terminal ER-lumen domain of Mmm1 among different fungal groups (Figure 2A, Figure S3). Several studies have shown that the ER domain of the *S. cerevisiae* protein is not essential for rescue of the growth and mitochondrial morphology defects seen in deletion mutants [9], [12], [16], [51]. However, these studies did not examine Tom40 assembly and it remains to be determined if changes in the ER-lumen domain affect the process of mitochondrial protein import/assembly in *S. cerevisiae*.

### Mutation of a conserved region of Mmm1 affects assembly of mitochondrial outer membrane proteins

**Figure 5:**
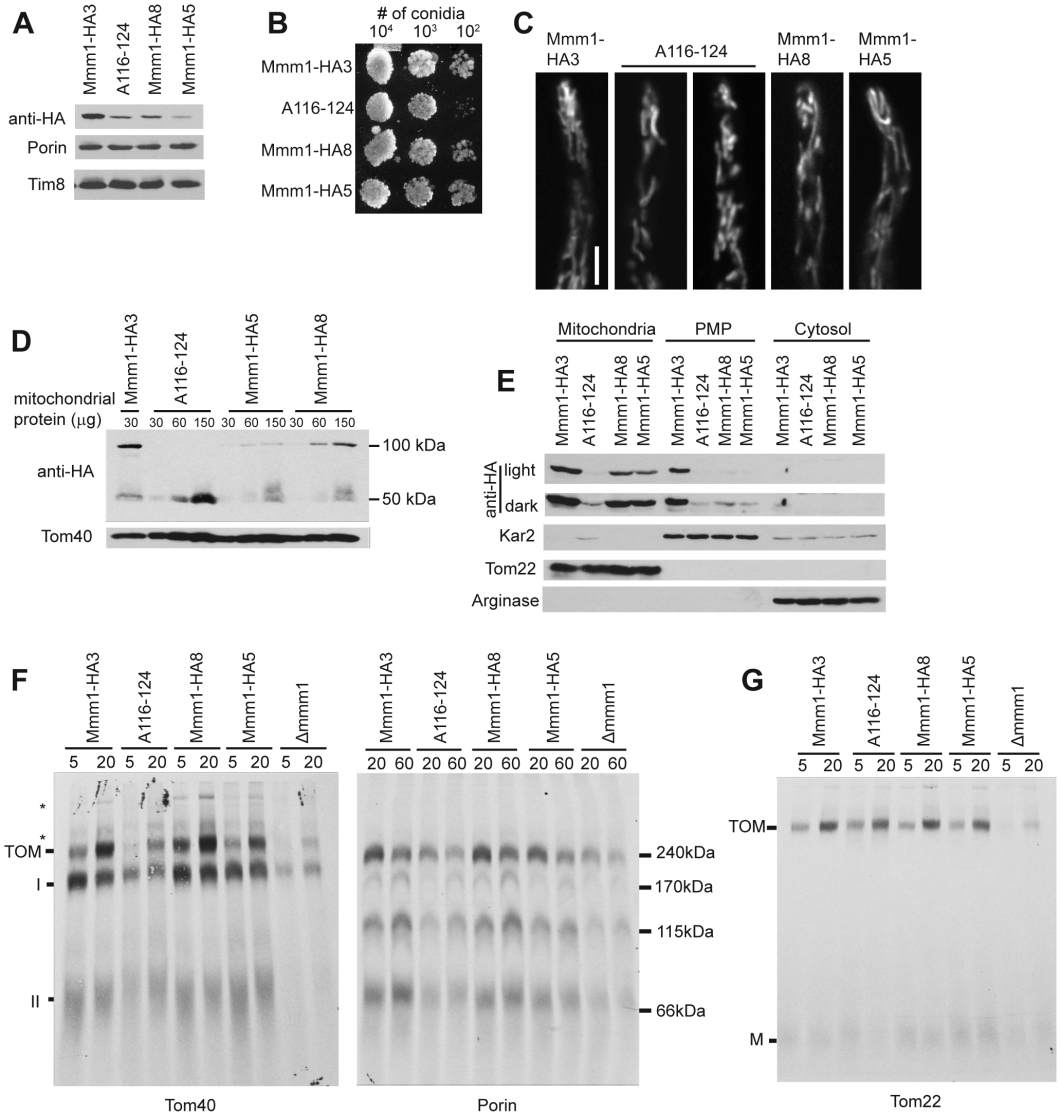
Characterization of *N. crassa* Mmm1 conserved region mutant A116-124. A. Western blot analysis of crude mitochondria (30 ug) from the indicated strains. Samples were subjected to SDS-PAGE, transferred to nitrocellulose and analyzed by Western blotting for the indicated proteins. B. Measurement of growth as in Figure 3C. C. Confocal microscopy of mitochondria as in Figure 3D. D. Non-reducing SDS-PAGE followed by Western blot analysis as in Figure 3E, but 30, 60, and 150 µg of mitochondria isolated from the indicated strains were loaded. E. Cell fractionation as in Figure 1A. F. Assembly of radiolabeled β-barrels as in Figure 4A using crude mitochondrial preparations as indicated in the Methods. G. Assembly of radiolabeled Tom22 as in Figure 4C.

The Mmm1 alignment revealed a region highly conserved in all fungi at position 116 to 124 of the *N. crassa* protein (Figure 2C and Figure S3). We constructed a mutant version of the HA-tagged *mmm1* gene in which all nine codons of the region were mutated to Ala residues. The mutant allele was transformed into *Δmmm1*. None of the resulting transformants contained the mutant form of the protein at the same level as the Mmm1-HA3 control strain in crude mitochondrial preparations (data not shown). Since the cassettes used for transformation were identical to those used to obtain Mmm1-HA3, except for the mutated region of *mmm1*, the decreased protein levels should not be due to decreased expression of the gene. We chose one strain (A116-124) for further analysis, but to allow functional assessment of the conserved region we required a strain with reduced levels of the HA-tagged wild type protein to serve as a control. Therefore, we re-examined strains from our original transformation of *Δmmm1* with plasmid pMmm1-HA to look for isolates with low expression of Mmm1-HA. Two strains, Mmm1-HA8 and Mmm1-HA5, were selected because A116-124 crude mitochondria contained levels of Mmm1HA intermediate between these two strains (Figure 5A).

Strain A116-124 exhibited a slight growth defect (Figure 5B) and contained mitochondria with a slightly larger diameter than those of the control strains (Figure 5C and Figure S2). When mitochondria isolated from these strains were subjected to non-reducing SDS-PAGE, Mmm1 in both low-expression control strains was found in the high molecular weight disulfide bonded form of the protein. However, in mitochondria from A116-124 no high molecular weight form could be detected (Figure 5D). Though it seemed unlikely, it was conceivable that the mutant protein was not properly integrated into the ER membrane. To test this possibility we performed alkali extractions on crude mitochondria from strain A116-124. The protein exhibited similar behavior to the control (Figure S5). Despite the fact that the mutant protein was readily detected in crude mitochondrial preparations (Figure 5A), only much reduced amounts were seen in flotation gradient purified mitochondria (compare ratios of Mmm1 in A116-124, Mmm1-HA8, and Mmm1-HA5 in the crude mitochondrial preparation of Figure 5A to those in the gradient purified mitochondrial fraction of Figure 5E). Thus, it appears that the A116-124 mutant form of Mmm1 is lost during flotation gradient mitochondria purification. Interestingly, in the strains containing lower levels of the wild type protein (Mmm1HA-5, Mmm1HA-8), Mmm1-HA is almost exclusively found associated with mitochondria rather than the ER containing PMP (Figure 5E). It is possible that when Mmm1 levels are low the protein may preferentially localize to mitochondrial attachment sites, whereas at higher levels additional Mmm1 might also localize to sites in the ER that are not involved in ERMES formation.

Mitochondria from A116-124 were impaired in their ability to assembly the β-barrel proteins Tom40 and porin to an extent midway between *Δmmm1* and the controls (Figure 5F). On the other hand, the assembly of Tom22 was not affected (Figure 5G). This suggests that regions of the Mmm1 protein differentially affect the assembly of TOB complex substrates.

### Mutants lacking Mmm2 exhibit a MOM protein assembly defect

**Figure 6:**
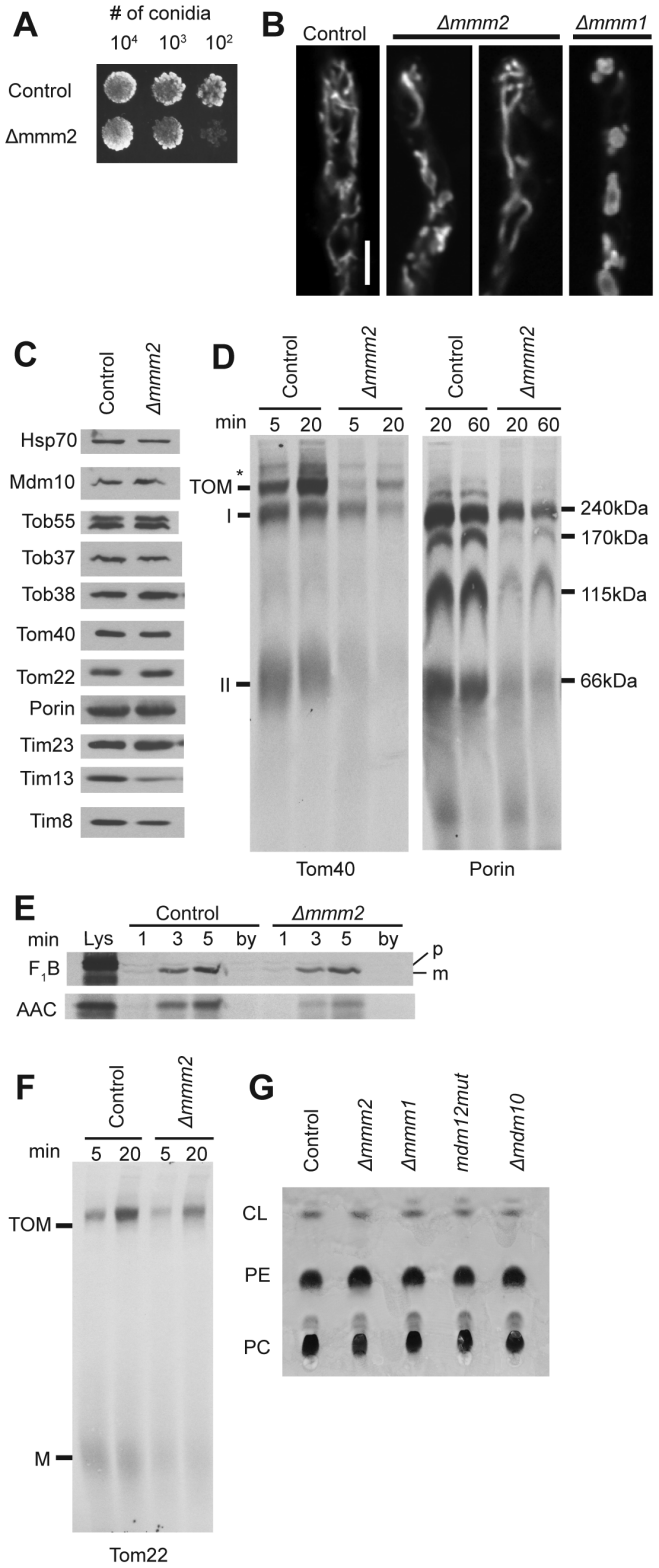
Characterization of the *mmm2* knockout strain. A. Measurement of growth rate as in Figure 3C. B. Visualization of mitochondria as in Figure 3D. C. Mitochondria (30 ug) isolated from the control and mutant strains were subjected to SDS-PAGE, transferred to nitrocellulose and analyzed on Western blots with antibodies to the indicated proteins (30 µg mitochondrial protein per lane). D. Assembly of radiolabeled β-barrel proteins Tom40 and porin as in Figure 4A and B, respectively. E. Radiolabeled F_1_β and AAC were imported into mitochondria isolated from the indicated strains. After import, the mitochondria were treated with proteinase K, reisolated, electrophoresed, transferred to nitrocellulose membranes, and examined by autoradiography. Lys, 33% of the input lysate containing radiolabeled protein used in each reaction; by (bypass import), mitochondria pretreated with trypsin to remove surface exposed receoptor proteins before 3 min of import with precursor proteins. This lane serves as a control to show no import occurs when mitochondrial surface receptors have been removed; p, preprotein; m, mature protein. F. Assembly of radiolabeled Tom22 as in Figure 4C. G. Total mitochondrial lipids were extracted from isolated crude mitochondria (300 µg protein) from the indicated strains in 1:1 chloroform : methanol. Lipids were then analyzed by TLC, stained with molybdenum blue, and photographed. PC, phosphatidylcholine; PE, phosphatidylethanolamine; CL, cardiolipin.

*Δmmm2* was identified in *S. cerevisiae* as a mitochondrial morphology mutant with a growth defect on non-fermentable carbon sources [15], [17]. The deletion mutant has also been shown to have decreased levels of phosphatidylethanolamine (PE) and cardiolipin (CL) [19]. Examination of a *Δmmm2 N. crassa* strain revealed a slight growth defect (Figure 6A) and the presence of enlarged mitochondria (Figure 6B and Figure S2). However, the mitochondrial morphology defect seen in *Δmmm2* is not as severe as that in *Δmmm1* (Figure 6B and Figure S2). Mitochondria isolated from *Δmmm2* contain wild-type levels of all mitochondrial proteins examined with the exception of the intermembrane space proteins Tim8 and Tim13 which are slightly reduced (Figure 6C). We have previously shown that these proteins are partially lost because of rupture of the MOM during the mitochondrial isolation procedure in cells containing enlarged mitochondria [26].

It has not yet been shown if lack of Mmm2 results in deficiencies of β-barrel assembly in *S. cerevisiae* or *N. crassa*. We examined the assembly of Tom40 and porin in mitochondria from a *Δmmm2* strain. The formation of both Tom40 intermediate complexes and the final TOM complex was reduced in *Δmmm2* mitochondria (Figure 6D). Similarly, formation of all porin import complexes in mitochondria from the mutant strain were reduced (Figure 6D). Import of the matrix targeted protein F_1_β into mitochondria isolated from *Δmmm2* was similar to the control, while import of the inner membrane targeted ATP/ADP carrier protein (AAC) was slightly reduced (Figure 6E). Since the small Tim proteins are involved in the import/assembly of both AAC and β-barrel proteins, it was conceivable that their reduced level could account for the observed deficiencies. However, we have previously shown that the loss of the intermembrane space small Tim proteins from other strains with enlarged mitochondria is due to increased breakage of the outer mitochondrial membrane during the isolation procedure. Artificially generating damaged wild type mitochondria allowed us to show that loss of the small Tim proteins was responsible for the import/assembly defects of AAC, but not the β-barrel proteins [26], [27]. We conclude that loss of Mmm2 results in the defects observed in the assembly of Tom40 and porin. Assembly of Tom22 was also moderately reduced in mitochondria lacking Mmm2 (Figure 6F). We also examined whole mitochondria for levels of CL, PE, and phosphatidylcholine (PC). No obvious deficiencies were seen in *Δmmm2*, or other ERMES component mutants (Figure 6G).

### Loss of Gem1 results in partial ERMES mutant phenotypes

**Figure 7:**
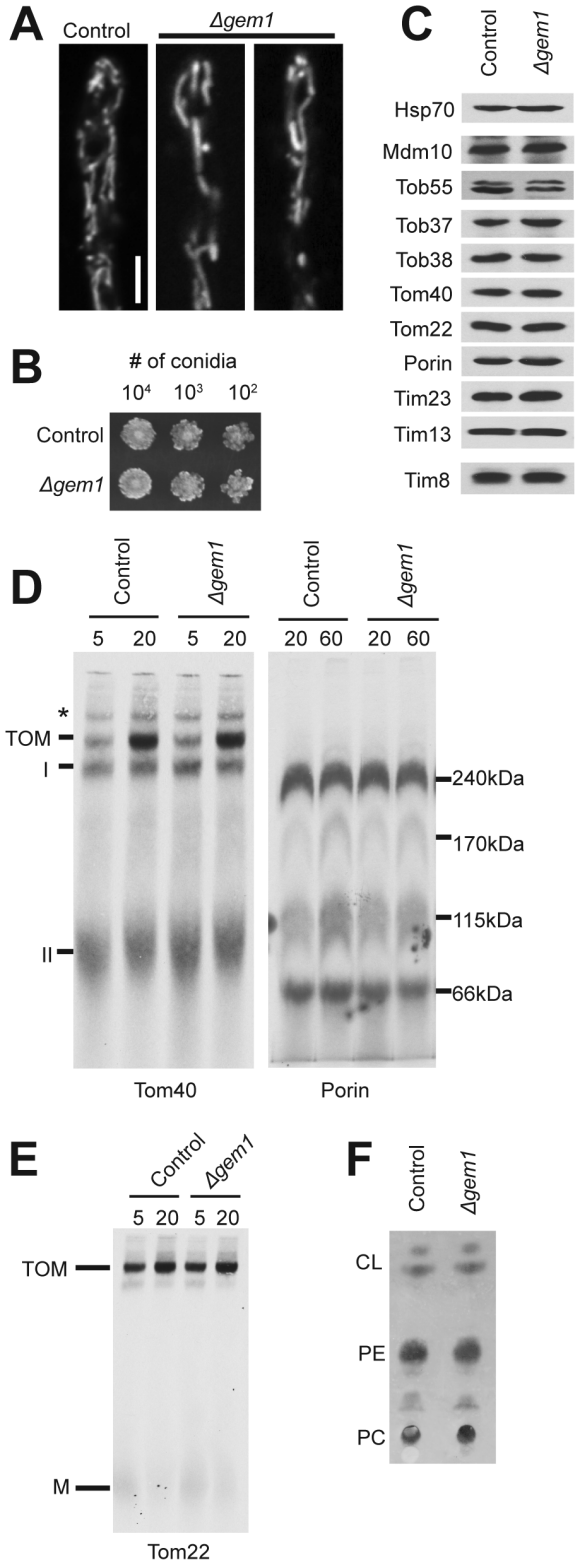
Characterization of the *Δgem1* strain. A. Examination of mitochondrial morphology as in Figure 3D. B. Growth rates as in Figure 3C. C. Steady state levels of mitochondrial proteins as in Figure 5A. D. β-barrel protein (Tom40 and porin) assembly as in Figure 4A and B, respectively. E. Tom22 assembly as in Figure 4C. F. Mitochondrial phospholipid content as in Figure 6G.

Gem1 mutants in *S. cerevisiae* exhibit some phenotypic characteristics of ERMES component mutants. They have altered mitochondrial morphology and delays in mitochondrial inheritance [59]. We analyzed a *N. crassa* strain lacking Gem1 and found a slightly larger mitochondrial tubule diameter (Figure 7A and Figure S2), but no alteration in growth rate (Figure 7B). Similarly, no changes in steady state mitochondrial protein levels (Figure 7C), assembly of mitochondrial outer membrane β-barrel proteins (Figure7D), assembly of Tom22 (Figure 7E), or mitochondrial phospholipid content (Figure 7F) were detected.

## Discussion

Our fractionation studies have shown that Mmm1 is a membrane anchored protein of the ER, in agreement with recent findings for *S. cerevisiae* Mmm1 [9], [11], [12]. This conclusion is supported by the observation that the Cys residue at position five of the protein is predicted to occur in the oxidizing environment of the ER lumen and is involved in disulphide bond formation. The C5S mutation results in loss of the disulphide bond and a Tom40 assembly defect. However, no other characteristics of strains lacking Mmm1 were observed in the C5S strain. This demonstrates that the set of typical phenotypes observed in cells lacking ERMES components can be separated, at least for Mmm1, and that defects in Tom40 assembly are not secondary to other aspects of the phenotype. Furthermore, since the *Δmmm1* strain has defects in Tom40, porin, and Tom22 assembly while the C5A mutation affects only Tom40, an effect on specific aspects of Tom40 assembly is implied for the Cys mutation.

The mechanism whereby a missing disulfide bond in the ER lumen affects Tom40 assembly in the MOM may be related to the structure and topology of Mmm1. Our findings suggest that disulphide bond formation results in an Mmm1 homodimer. Dimerization in the ER lumen would keep two subunits in close proximity. Subunits acting in tandem may be important for interaction with other ERMES components or other possible Mmm1 functions. A problem in assessing how Mmm1 could directly influence TOB complex function is based on simple physical constraints. ERMES in *S. cerevisiae* are reported to occur in only a small number of punctae in fluorescence microscopy studies [9], [11], whereas the TOB complex would be expected to have a more random distribution throughout the MOM. However, the distribution of the ERMES and its components has not been investigated in *N. crassa* and it is conceivable that the arrangement is different in the organism. Furthermore, it has been recently suggested that there may be many smaller ERMES in *S. cerevisiae* than previously thought [10].

The phenotype of the A116-124 mutant is complex. No transformants expressing the mutant protein at the level of the control HA-tagged Mmm1 protein could be identified. Furthermore, the amount of Mmm1 in crude mitochondrial preparations from the mutant strain is reduced even further upon gradient purification of mitochondria. This may be due to reduced stability of the protein and/or reduced binding to mitochondria. Surprisingly, even though the A116-124 mutation occurs in the cytosolic domain of Mmm1, it eliminates disulphide bond formation in the ER lumen domain. Perhaps conformational changes in the cytosolic domain are communicated through the ER membrane to the lumen domain, preventing Cys bond formation. Despite the much reduced level of the protein observed in purified subcellular fractions, it still partially rescues the mitochondrial morphology, Tom40 assembly, and porin assembly defects seen in the *Δmmm1* strain, while fully rescuing the Tom22 assembly defect.

Although Mmm2 has been recognized as an ERMES component, it has not been previously examined with respect to mitochondrial import/assembly. Here we show that deletion of the gene results in a defective assembly phenotype for Tom40 and porin that resembles the defects seen in *mmm1* or *mdm12* mutants of *N. crassa*. We have also shown that mitochondria isolated from cells lacking any of the ERMES structural components are defective in the assembly of Tom22. This differs from *S. cerevisiae* where mitochondria lacking Mdm12 were shown to assemble Tom22 normally [25].

The mechanisms by which ERMES components affect the assembly of TOB complex substrates are not known, though models exist for the role of Mdm10 in Tom40 assembly. One suggests that Mdm10 is involved in the release of the Tom40 precursor from the TOB complex [34]. This model is based on the observations that both deficiency of Mdm10 or overexpression of Mdm10 result in Tom40 assembly defects, while only overexpression results in a porin assembly defect. It was suggested that Mdm10 competes with β-barrel precursors for binding sites on the TOB complex and that the precursors have different affinities for these sites. Tom40 can be detected in association with the TOB complex as intermediate I during assembly assays, implying a relatively strong association. The porin precursor is not detected as such an intermediate in *S. cerevisiae*, implying weaker binding. Thus, Mdm10 is required to release Tom40, but not porin, from its binding site on the TOB complex. On the other hand, overexpression of Mdm10 inhibits assembly of both precursors because the precursor binding site on the TOB complex is blocked by excess Mdm10. We previously suggested that *N. crassa* Mdm10 has a general effect on TOB complex function [26]. Interestingly, our model fits with certain aspects of the one described above since *N. crassa* strains lacking Mdm10 do have a porin assembly defect [26] and a stable intermediate for porin assembly can be detected in *N. crassa* [36]. A third model suggests that the role of Mdm10 is to assist the TOB complex with the assembly of Tom22 [29], [30]. Tom40 assembly is affected in mitochondria lacking Mdm10 because Tom22 is necessary for assembly of Tom40 into the TOM complex [29], [60]. This model seems unlikely to be true for *N. crassa* since both porin and Tom40 assembly are reduced in mitochondria lacking Mdm10 and there is no known dependence on Tom22 for porin assembly. Very little is known about the roles of other ERMES components. However, recent findings describing complex interactions between ERMES, TOB, and mitochondrial inner membrane complexes controlling mitochondrial architecture may influence the function of all the components involved [61], [62], [63], [64], [65], [66], [67].

Mutants lacking Gem1 in *S. cerevisiae* contain large globular mitochondria, exhibit a growth defect on non-fermentable carbon sources [59] and are delayed in the transfer of mitochondria to daughter buds [68]. The Gem1 protein is well conserved among eukaryotes and contains two GTPase and two calcium binding EF-hand domains [69]. There is debate as to whether *S. cerevisiae* Gem1 is involved in ERMES function or regulation. Two groups have identified Gem1 as a member of the ERMES and it was suggested that the protein plays a role in the regulation of the size and number of ERMES complexes [11], [12]. However, another group could not find evidence of Gem1 associating with the ERMES and found no alterations in ERMES size or numbers in strains lacking the protein [10]. Here we have shown that while the *Δgem1* mutant has a minor mitochondrial morphology defect in *N. crassa*, other ERMES phenotypes, such as inefficient β-barrel and Tom22 assembly, are absent. If Gem1 is an ERMES member in *N. crassa*, its role is different than the other proteins in the complex.

## Supporting Information

Figure S1 **Mmm1-HA rescues** ***Δmmm1*** **phenotypes.**
(PDF)

Figure S2 **Measurement of mitochondrial diameter in mutant strains.**
(PDF)

Figure S3 **Alignment of fungal Mmm1 proteins.**
(PDF)

Figure S4 **Cysteine mutants have normal TOM complex and Tim8/13 levels.**
(PDF)

Figure S5 **Alkali extraction of mitochondria from Mmm1 mutant A116-124.**
(PDF)
