# Supplementary material for: Analysis of Mutations in Neurospora crassa ERMES Components Reveals Specific Functions Related to β-Barrel Protein Assembly and Maintenance of Mitochondrial Morphology
Source: PLoS One. 2013 Aug 5;8(8):e71837. doi: 10.1371/journal.pone.0071837 (PMC3733929; doi:10.1371/journal.pone.0071837)
Supplement: Figure S1 — Mmm1-HA rescues Δmmm1 phenotypes. (PDF) [file pone.0071837.s001.pdf]

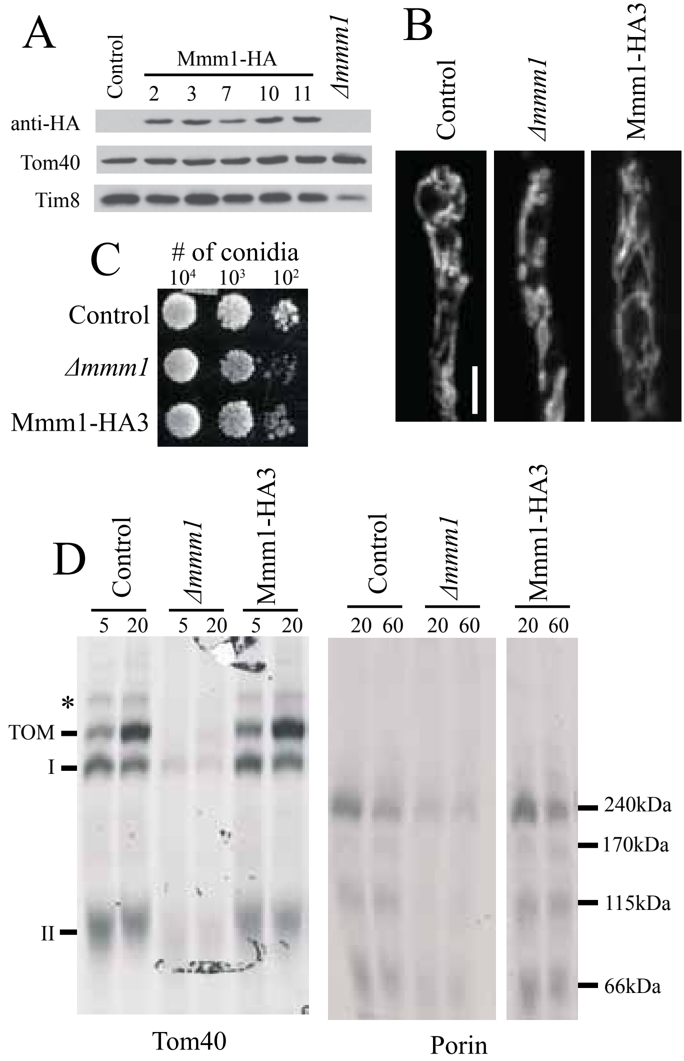

SUPPORTING INFORMATION FIGURE S1. Mmm1-HA rescues *Δmmm1* phenotypes. A. Mitochondria isolated from the indicated transformant strains were subjected to SDS-PAGE and analyzed by Western blot for detection of the indicated proteins. B. The control *Δmmm1* and Mmm1-HA3 strains were grown on solid Vogel's media, stained with MitoTracker Green FM and examined by confocal fluorescence microscopy. Bar indicates 10 μm C. Conidiaspores from the indicated strains were spotted as tenfold dilutions on Vogel's sorbose medium plates. Plates were incubated at 30°C for 48h. D. Assembly of Tom40 and Porin. Radiolabeled precursors were incubated with isolated mitochondria for the indicated times. Mitochondria were then reisolated and dissolved in 1% digitonin. The samples were subjected to BNGE, transferred to PVDF membrane and analyzed by autoradiography. The mature TOM complex and intermediates I and II are indicated. The four major complexes containing radiolabeled porin are indicated by their estimated molecular masses.
