# Supplementary material for: Analysis of Mutations in Neurospora crassa ERMES Components Reveals Specific Functions Related to β-Barrel Protein Assembly and Maintenance of Mitochondrial Morphology
Source: PLoS One. 2013 Aug 5;8(8):e71837. doi: 10.1371/journal.pone.0071837 (PMC3733929; doi:10.1371/journal.pone.0071837)
Supplement: Figure S2 — Measurement of mitochondrial diameter in mutant strains. (PDF) [file pone.0071837.s002.pdf]

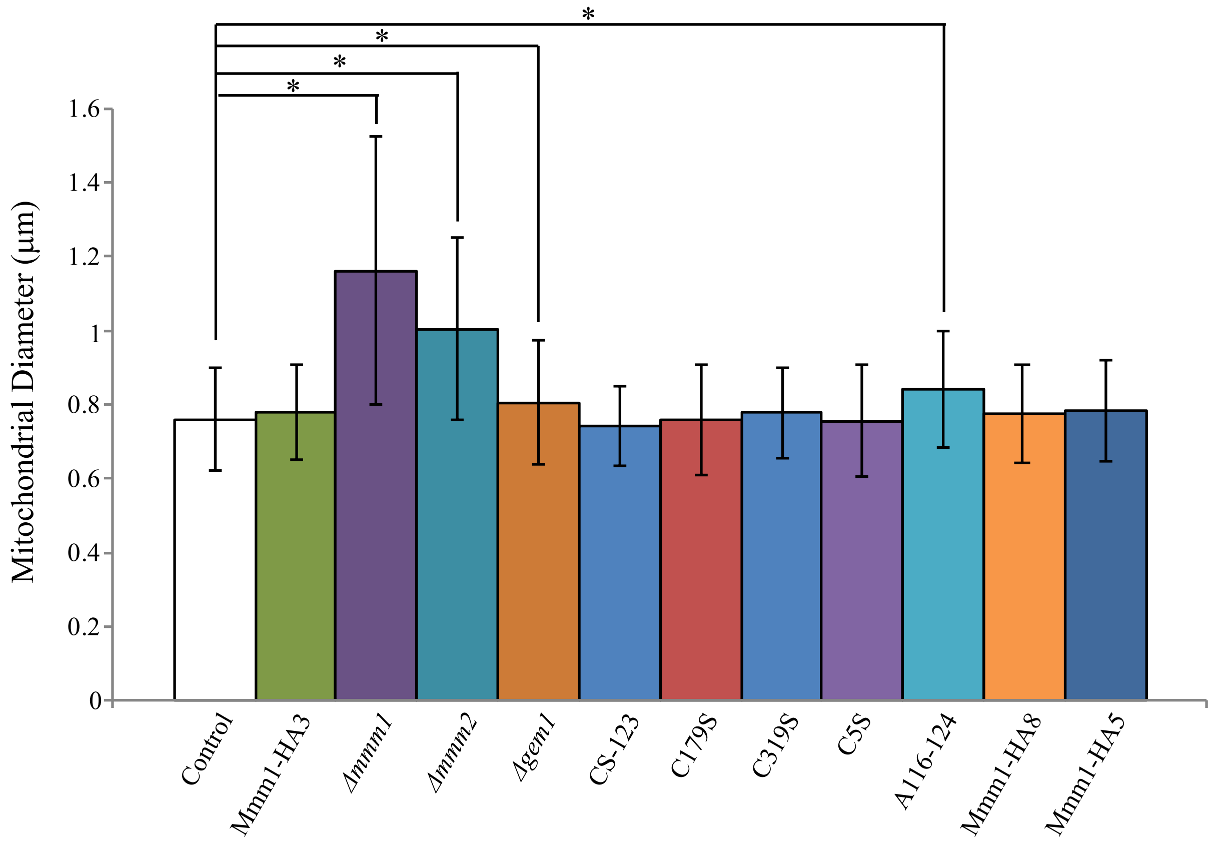

SUPPORTING INFORMATION FIGURE S2. Measurement of mitochondrial diameter in mutant strains. Mutants lacking Mmm1, Mmm2, Gem1, or expressing the A116-124 Mmm1 mutant contain mitochondria with larger diameters than other strains. Mitochondrial diameter was measured using Metamorph offline software. Measurements were obtained for all visible mitochondria from at least 4 hyphal tips for each experiment. When measuring longer mitochondria, measurements were taken approximately every micrometer. The mean hyphal widths of control and experimental populations were compared via a two-tailed Student's t-test assuming unequal variances at $p= 0.01$. Bars indicate standard deviation. Asterisks indicate significant difference $P < 0.01$
