# Supplementary material for: Analysis of Mutations in Neurospora crassa ERMES Components Reveals Specific Functions Related to β-Barrel Protein Assembly and Maintenance of Mitochondrial Morphology
Source: PLoS One. 2013 Aug 5;8(8):e71837. doi: 10.1371/journal.pone.0071837 (PMC3733929; doi:10.1371/journal.pone.0071837)
Supplement: Figure S3 — Alignment of fungal Mmm1 proteins. (PDF) [file pone.0071837.s003.pdf]

SUPPORTING INFORMATION FIGURE S3. Alignment of fungal Mmm1 proteins. Conserved cysteines are shaded in grey. A116-124 is shaded in grey in the *N. crassa* protein. The membrane spanning domain of the *N. crassa* protein is underlined and shaded in grey. Residues in the C.g. sequence resulting from a potential missed intron are highlighted. Abbreviations: N.c., *Neurospora crassa*; G.z., *Gibberella zeae*; C.g., *Chaetomium globosum*; S.m., *Sordaria macrospora*; M.o., *Magnaporthe oryzae*; P.a., *Podospora anserina*; F.o. *Fusarium oxysporum*; V.d., *Verticillium dahliae*; A.n., *Aspergillus nidulans*; *Trichophyton tonsurans*; C.i., *Coccidioides immitis*; P.b., *Paracoccidioides brasiliensis*; S.s., *Sclerotinia sclerotiorum*; B.f., *Botryotinia fuckeliana*; P.t., *Pyrenophora teres*; S.c., *Saccharomyces cerevisiae*; *Kluyveromyces lactis*; C.a., *Candida albicans*; S.p., *Schizosaccharomyces pombe*; U.m., *Ustilago maydis*; C.n., *Cryptocuccus neoformans*; R.o., *Rhizopus oryzae*; B.d., *Batrachochytrium dendrobatidis.*

```
N.c.            ------------------------------------------------------------
G.z.            ------------------------------------------------------------
C.g.            ------------------------------------------------------------
S.m.            ------------------------------------------------------------
M.o.            ------------------------------------------------------------
P.a.            ------------------------------------------------------------
F.o.            ------------------------------------------------------------
V.d.            ------------------------------------------------------------
A.n.            ------------------------------------------------------------
T.t.            ------------------------------------------------------------
C.i.            ------------------------------------------------------------
P.b.            ------------------------------------------------------------
S.s.            ------------------------------------------------------------
B.f.            ------------------------------------------------------------
P.t.            ------------------------------------------------------------
S.c.            -----MTDSENESTETDSLMTFDDYISKELPEHLQRLIMENLKGSTTNDLKQTSNNSEFN 55
K.l.            MEMSELLASEVVSSGPDYAKKSVDGLNMTAANGTNDTLMT-LDEYLNKSLPLHLEQLILD 59
C.a.            -----MSQDLIETTATTTKIVEAR----ELGHQIHDSLLEQLKLQQ-EELLQQQRDLFFQ 50
S.p.            ------------------------------------------------------------
U.m.            ------------------------------------------------------------
C.n.            ------------------------------------------------------------
R.o.            ------------------------------------------------------------
B.d.            ------------------------------------------------------------


N.c.            ---------------MADICPSRSEP-------------------------TLSFTQGLI 20
G.z.            --------------MAGDTCPQPTEP-------------------------TLSFTQGLI 21
C.g.            --------------MARDVCPTNSEPTVSRRVVPTLLRGWDVNVANCRWVYRLSFTQGFI 46
S.m.            ---------------MADICPARSEP-------------------------TLSFTQGLI 20
M.o.            --------------MTPDSCPVRPEP-------------------------TLSFTQGLI 21
P.a.            --------------MAQDVCPTRSEP-------------------------SLSFLQGLI 21
F.o.            --------------MEADTCPRLIEP-------------------------TLSFTQGLI 21
V.d.            --------------MADKTCPATSEP-------------------------TLSFAQGLV 21
A.n.            -----MAFQQGTPGPPVELCSTFTDHDE--------------------TVSSLSFTQGFL 35
T.t.            -----------MSSPENASCP--------------------------PPQHSLSFTQGLL 23
C.i.            ---------------MSN--DTSAQ----------------------PAQSSLSFTQGLL 21
P.b.            ---------------MAGSTSASLQTPYFPSSTQINPVRVDHTLPLPPSQPSLSFTQGLL 45
S.s.            ------------------MWLDDVAS-------------------------ELSFTQGLL 17
B.f.            -------------MTIPAPIPDKAES-------------------------SLSFTQGLL 22
P.t.            -----------MAEEVPTAVPLATPAGSS----------------------SLSFTQGFL 27
S.c.            VSKNGSFKGLDDAIQALQMQSVLHPS-------------SLGSLATSSKFSGWSFAQGFF 102
K.l.            ANQKELFDSAAKSLLSSTLLAKQQQSL------------QIAPIQPQSSFSSQSFAEGLI 107
C.a.            EQQ--------------LQLQQVTQP-------------------VSNNGNTWSFTQGLV 78
S.p.            -------------------MIHLPQG---------------------------SFTQGLI 14
U.m.            ---------------MQQPQQQDLQIGLPYAPVQPPIPSPAAYFAYLPSPSRWTFTQGLI 45
C.n.            --------------------------------------------MSETFSPNLTFTEGFV 16
R.o.            ------------------------------------------------------------
```

```
B.d.            ------------------------------------------------------------

N.c.            LGQLSVVLLLAAFIKFFIFGDPPSPEVVASIRATDRRSRTLAHKKS------ILSLRETN 74
G.z.            VGQLSVVLVLAAFIKFFIFGDPPSPDVTASLRATERRSRTLAHKRS------LLSLRSPG 75
C.g.            LGQISVVLLIAAFIKFFIFGDPPSPEITASIRAAERRSRTLAHKRS------LLSLRESS 100
S.m.            LGQLSVVLLLAAFIKFFIFGDPPSPEVVASIRATDRRSRTLAHKKS------ILSLRETN 74
M.o.            VGQISVVFLIAAFIKFFIFGDPPSAEETASLRASERRSRTLAHKKS------LLSLRTSN 75
P.a.            LGQLSVVLLIAAFIKFFIFGEAPSAEETASIRATERRSRTLAHKKS------LLSLRSAA 75
F.o.            VGQLSVVLVLAAFIKFFIFGDPPSADVTASLRATERRSRTLAHKRS------LLSIRSPA 75
V.d.            LGQLSVVLVLAAFIKFFIFGDPPSPEVTASLRATERRSKTLAHKKS------LVGLRAAT 75
A.n.            LGQLSVVLLIGAFIKFFIFGEAPPPPSRG--LRASTHRRSNSIFS--QDAPPPRSLREKP 91
T.t.            LGQLSVVLLIGAFIKFFIFGEAPSSSSRGISQRAAPRKRSYSANSTLFRDAASRSLKESA 83
C.i.            VGQLSVVLLIGAFIKFFIFGEASPSSSRSQTRRTSPHKRSYSISG--ARDLGSRSLKEKP 79
P.b.            VGQLSVVLLIGAFIKFFIFGEAPPPPSRGLSNRTSTHPRSYSINAA-STDSSPRPLREKP 104
S.s.            LGQLSIVILIGAFIKFFIFGDPPSPDVSAALRATERRSRTLAHKRS------LLTLRSST 71
B.f.            LGQLSIVILIGAFIKFFIFGDPPSPDVTAALRATERRSRTLAHKRS------LLTLRSST 76
P.t.            LGQLSIAILIFCFIKFFIFGEPPSADDRALHLNSLRRARTLAHQQS------YKQLQTRA 81
S.c.            VGQLSIVLLFIFFLKFFIFSDEPSKSKNPKPAASRHRSKFKEYPFI-----------SRE 151
K.l.            VGQLSVIVALIFVIKFFVFSEGGTKTATAKSVGS--ASSFMDS--T-----------KNS 152
C.a.            IGQVSVIFIIIVFVKFFVFADSSSHIPTKPGLDG--ATGVIVK--------------RNK 122
S.p.            VGQLLTLAIIYVFLRFFLFCSPIPKSVANSPKQTGNETPDETPSTP-------------- 60
U.m.            VGQVSMVIVALLLIRYVIFEDSATALEKERLMRLKVSQRRSKLHAKALLQDARKANSAAA 105
C.n.            LGQASFLIIILLLFIRYVVFSPSEQIDHEG--------------------------WRKRR 50
R.o.            ----------MLLVQKARYIPSRPSLN------------------------------TAP 20
B.d.            --------------MHQRIAVSKPK--------------------------------PVS 14

N.c.            ALQLVQNP----ALNKKH--VLRPG---PPILTIGSILSKTYYKVDSHQPESLDWFNVLI 125
G.z.            NR----QDR---ELNRKKSTVLRN----PPALTIGSILSKTYYNVDSHQPESLDWFNVLI 124
C.g.            TRRAGQQP----TLNRKKSSILRPS---PPPLTIGSILDKTYYKVDSHQPESLDWFNVLV 153
S.m.            ALQLVQNP----ALNKKH--VLRPG---PPTLTIGSILSKTYYKVDSHQPESLDWFNVLI 125
M.o.            QRPGSQQQQS--VLNRKKSSILRSG---PPSLTIGSILNKTYYRVESHQPESLDWFNVLV 130
P.a.            TQRQGSQPPALPALNKKKSSILRSN---PPTLTIGSILDKTYYKVDSHQPESLDWFNVLI 132
F.o.            NRGDRSQDR---SLSRKKSTVLRN----PPTLTIGSILSKAYYNVDSHQPESLDWFNVLI 128
V.d.            GR---PGQP---SLNKKKSSVLRS----PYNLTIGSILSKTYYNVDSHQPESLDWFNVLV 125
A.n.            STSNVLRPVP--SSATNTRSILRKTYYSAIPPNPS---SKHRIHHSSHQPESLDWFNVLI 146
T.t.            S-SNVLRPVP--SSSTNTKSILRKTYYNAIPTNFQ-KNGRNRLHHSTHQPESLDWFNVLI 139
C.i.            S-SNVLRPVP--SSSTNTRSILRKTYYSANPTNFTSKHGRHRPHHSTHQPESLDWFNVLI 136
P.b.            STSNILRPVP--SSSTNTRSILRKTYYSATPTHPTPKHGRPRLYHSSHQPESLDWFNVLI 162
S.s.            PRHASQS------LNRKRSSVLRN----PAPLTTNAILSKTYYNVDSHQPESLDWFNVLI 121
B.f.            PRRASQP------LNRKRSSVLRN----PAPLTTNAILSKTYYNVDSHQPESLDWFNVLI 126
P.t.            NSTSLSLR------HKPSTSIIRKGEETRGGPSIATILAKTYYNVKGHQPESLDWFNVLI 135
S.c.            FLTSLVRKGA------KQHYELNEEAENEHLQELALILEKTYYNVDVHPAESLDWFNVLV 205
K.l.            ILSTIIKRGG------KDGLEVDDK-DNEKSRQINSILEKTYYNVETHSPESLDWFNVLI 205
C.a.            NKKHSNGQFA------NDGENEDDTSLDSNQSKISSILEKTYYDVNNHASESLDWFNVLV 176
S.p.            -------------------LSNNKKRYKKPLTILEPHILNLLYDVNEHEPESLDWFNVLI 101
U.m.            SAAAAAAPSP----ASHPLRKSHRLASDTRASMFANILDKTAYDLSSHLPESADWLNVMF 161
C.n.            AERADLLSN-------------------HTPPPLSNLLSKTSYDMSIHPAESSDWVNVLL 91
R.o.            SVAAAALPN-------------------------DHITLKTYYDVIHHPPESTDWLNVLL 55
B.d.            AQQRRTIDS-------------------------LILGKIGYNTSQHPFESCNWLNILI 48
                                                        .      *  ** :*.*::.

N.c.            AQTIAQFRSDA-----QHDDAILSSLSKALNG--------------------TARPDFLD 160
G.z.            GQTIAQFRSDA-----QHDDAILDSLSKALNG--------------------GSRPDFID 159
C.g.            AQTIAQFRSDA-----QHDDAILTSLTKALNG--------------------TSRPDFVD 188
S.m.            AQTIAQFRSDA-----QHDDAILSSLSKALNG--------------------TARPDFLD 160
M.o.            AQTIAQFRSDA-----QHDDAILTSLTKALNG--------------------TSRPSFLD 165
P.a.            AQTIAQFRSDA-----QHDDAILTSLSKTLNG--------------------TSRPDFVD 167
F.o.            AQTIAQFRSDA-----QHDDAILDSLTKALNG--------------------DSRPDFID 163
V.d.            AQTIAQFRSDA-----QHDDAILSSLTKALNG--------------------TSRPDFVD 160
A.n.            AQTIAQYRQTAYLLKDSPTSSILHSLTAALNNP-------------------EKKPSFID 187
T.t.            AQLIAQYRQTAYLLKDSPTSSILDSLTDTLNNV-------------------EKKPSWID 180
```

```
C.i.           AQTIAQYRQTAYILKDSPTSSILESLATTLNNP-------------------EKKPSFID 177
P.b.           AQTIAQYRQTAYILKDSPTSSILASLSETLNNP-------------------EKKPSFID 203
S.s.           AQTIAQFRADA-----QHDDAILTSLTKALNG--------------------GNRPDFLD 156
B.f.           AQTIAQFRADA-----QHDDAILTSLTKVLNG--------------------GNRPDFLD 161
P.t.           AQTIAQLRADA-----RQDDAILTSLTEVLNT--------------------GSKPDWIG 170
S.c.           AQIIQQFRSEA-----WHRDNILHSLNDFIGRKS------------------PDLPEYLD 242
K.l.           AQTIHQFREEA-----LQKNNILNSLNDFIERRS------------------NELPQYLD 242
C.a.           AQTISQLRSEA-----LLKDNIYHSLNNFLTN--------------------AKLPDFID 211
S.p.           AQALIQFRYDA-----CSNDVALRKLETVLNKG-------------------AQDKSMVD 137
U.m.           AQAIAGYREDVLTGG--VSSHHHTASDAIPSPNPLEPQKERTARDLMEEILNRATSSFLD 219
C.n.           AQILQGYRNDLLSEG--GEEGARQRIEGWLNPK-------------------GENLSWLD 130
R.o.           AQVILQYRQDAS-----INNRMSCALDSVFNSG--------------------VRPSFVG 90
B.d.           AQFLLTLRTDAEFG-----LKSVSMLDSILNSA--------------------WKPSFLG 83
               .* :   *                                                . :.

N.c.           EIKVTELSLGEDFPIFSNCRIIPVDEDGLSFGTGKAFDANMATREGARLQARMDVDLS-D 219
G.z.           EIRVTELSLGEDFPILSNCRIIPVDEDGVSLGHGKKFDPDKAARDGTRLQARMDVDLS-D 218
C.g.           EIRVTELSLGEDFPIFSNCRIIPVDEDGLNLQSGKGLDANMATREGARLQARMDVDLS-D 247
S.m.           EIKVTELSLGEDFPIFSNCRIIPVDEDGLSFGTGKAFDANMATREGARLQARMDVDLS-D 219
M.o.           EIRVTELSLGEDFPIFSNCRIIPVDEDGLDFGPGKAFDANMATRDGGTLQARMDVDLS-D 224
P.a.           EIRVSELSLGEDFPIFSNCRIIPVDEDGLQFGAGKAFDPKQAAREGARLQARMDVDLS-D 226
F.o.           EIRVTELSLGEDFPILSNCRIIPVDEDGVSLAPGKKFDPATAARDGLRLQARMDVDLS-D 222
V.d.           EIRVTKLSLGEDFPIFSNCRIIPVDEDGLSLGNSKTIDASAATREGAKLQARMDVDLS-D 219
A.n.           KITVTDISLGEEFPIFSNCRIIAVDDP---------------MSDGGRLQALLDVDMSDD 232
T.t.           RINVTDISIGEEFPIFSNCRVIAVDDP---------------NSDGGRLQALMDVDLSDD 225
C.i.           DITVTDISLGEEFPIFSNCRVIAIDDP---------------SSDGGRLQALMDVDLSDD 222
P.b.           IIKVTDISLGEEFPIFSNCRVIAVEDP---------------NSDGGRLQALMDVDLSDD 248
S.s.           EIKVTELSLGEDFPIFSNCRVIPVDEDGITLGR----EGGAAGREHGRLQARMDVDLS-D 211
B.f.           EIKVTELSLGEDFPIFSNCRVIPVDEDGMTLGR----EGGAAGREHGRLQARMDVDLS-D 216
P.t.           EIKVTEIALGDEFPIFSNCRVMPA-EDGFWYGPG------NTGNDKERLQARMDVDLS-D 222
S.c.           TIKITELDTGDDFPIFSNCRIQYSP-----------------NSGNKKLEAKIDIDLN-D 284
K.l.           QIKITEVDIGDDFPIFSNCIIQYSP-----------------NSNKKRLEAKIDIDLS-D 284
C.a.           TINLTEIDIGDDFPIFSNCRIKYG-------------------EDLKRLEAKIDVDLS-D 251
S.p.           HIYVRDLSLGDGFPVFSHCRVLPHQHN-------------------SSQLRAEMLVSLT-D 178
U.m.           PIRVTEADFGDAYPIFTNARVRPADDT--------------------GRTRIEIDVDYS-D 259
C.n.           PIDVTSLSLGTSYPLLSNARIRPADGQ--------------------GRLRAEIDVDYL-D 170
R.o.           PIHVTELNLGQEFPIFSRARIRPSDEA--------------------GSTRAEIDFEYS-D 130
B.d.           DISITNFSLGEEYPTLKNARVRFAEPE--------------------SGMKIHVDFSFD-D 123
                * : .   *  :* :... :                            .  : ..   *

N.c.           MITLAVETKLLLNYPKRLSAVLPVALAVSVVRFSGTLSISFIPSN--------------- 264
G.z.           MLTLAVETKLLLNYPKKLSAVLPVALAVSVVRFSGTLSISFIPSN--------------- 263
C.g.           MITLAVETKLLLNFPKRLSAVLPVALAVSVVRFSGTLSISFVPSN--------------- 292
S.m.           MITLAVETKLLLNYPKRLSAVLPVALAVSVVRFSGTLSISFIPSN--------------- 264
M.o.           MITLALETKLLLNYPKKLTAVLPVALAVSVVRFSGTLAISFIPSN--------------- 269
P.a.           MITLAVETKLLLNFPKRLSAVLPVALAVSVVRFSGTLSISFNPSN--------------- 271
F.o.           MLTLAVRTKLILNYPKKLSAVLPVELAVSVIRFSGTLSISFIPSN--------------- 267
V.d.           LLTLALETKLLLNYPKKLSAVLPVALTVSVRRFSGTLSVSFIPSN--------------- 264
A.n.           NLSIAVETSLVLNYPKPCSAILPVALSISVVRFSGTLCISLVPAS--------------T 278
T.t.           NLSLAIETNLLLNYPKPASAVLPVALSVSVVRFSGTLCISFVPSP--------------G 271
C.i.           NLSLAIETNLVLNYPKPYSAILPVALSVSVVRFSGTLCISFVPGT--------------T 268
P.b.           NLSLAIETSLLLNYPKPFSAVLPVALAVSVVRFSGTLCISFVPGP--------------R 294
S.s.           FITLAVETKLLLNYPKPLVAVLPVALAVSVMRFSGTLSISFVPGS--------------- 256
B.f.           FITLAVETKLLLNYPKPLVAVLPVALAVSVVRFSGTLSISFVPGS--------------- 261
P.t.           VITIGVETTLNLNWPKPMSAVLPVALAVSIVRFSGTLAMSFIPSSSPPSTTAPMPSPTCN 282
S.c.           HLTLGVETKLLLNYPKPGIAALPINLVVSIVRFQACLTVSLTN----------------- 327
K.l.           RLALGIETKLLLNYPKPFSAALPIKLTVSIVRFQACLTVSLTT----------------- 327
C.a.           TLTLGIATKLLLNQPRPLTAVLPVSMTVSIVRFSGCLTVSLINTKDIDLKNVDKTSNMNG 311
S.p.           NINCTVDTKLLLNFPKPAFATLPLSITVRICKFVGKVSLFTTLIVYS------------- 225
U.m.           QITLAIDTKLLINFPKPRFAVLPVSLGLTIVRFSGTLAIELFSSD--------------- 304
C.n.           SLSMTLSTAVLVNFPKPRFAVLPVTLGVELVSIGGTMSVQLHEPI--------------- 215
R.o.           QVTLGIETQLILNWPKQAFAVLPVSLVLSVVRFSGTLTIELINP---------------- 174
B.d.           QLTLGIDTQMLINYPKPGMAALPISIVLSIVKFSGTFVIEFVSKP--------------- 168
```

```
                  :   : * : :* *:   * **: : : :  : . . :

N.c.            ---------PSNNE-------------------------------------------PAK 272
G.z.            ---------PSQST-------------------------------------------PTR 271
C.g.            ---------PSENT-------------------------------------------PTK 300
S.m.            ---------PSNNE-------------------------------------------PAK 272
M.o.            ---------PSQST-------------------------------------------PTK 277
P.a.            ---------PSENT-------------------------------------------PTK 279
F.o.            ---------PSQST-------------------------------------------PTR 275
V.d.            ---------PSQST-------------------------------------------PTM 272
A.n.            PPLHTPSPMPSPPTAGAQPAAGAQPTDGGDIP----PKSSS----------------KSN 318
T.t.            TTS----ESSSNPPPHSEGQNEP---RPGQED----PRRPSSKDGIRS------GIPKTS 314
C.i.            QTSTHLATSPSNIDPTLQTNDYSGANRRGNRR----QERTDTEQATQANNAGTTGIPKTS 324
P.b.            TSDQTMSPIPTPHDTTSEAIDDQSSDQPSPAQNPDGPKDAHANTSNTTDASSKHGIPKTS 354
S.s.            ---------PLNGS-------------------------------------------PTT 264
B.f.            ---------PLNGS-------------------------------------------PTT 269
P.t.            THRSSSPSRPTSSSGAPP-------------------------------------HRPTT 305
S.c.            --AEEFASTSNGSSSENGMEGNS----------------------------------GYF 351
K.l.            --DEQFVPTSEETN-DDEMGNDK----------------------------------GYY 350
C.a.            YSKENANGDGASSSNNDEDEDDG----------------------------------GTA 337
S.p.            ---LYDSLHPSPGGLKHQVLNRITVIMIYFSP-------------------SNGAGQPAY 263
U.m.            -------------------------------------------------------PNATV 309
C.n.            -------------------------------------------------------EDRQH 220
R.o.            -------------------------------------------------------PETTT 179
B.d.            -------------------------------------------------------ILPVC 173

N.c.            MIFTFLDDYRLDFSIRS------------------------LLGSRSRLQDVPKIAQLVE 308
G.z.            MIFNFLDDYRLDFSIRS------------------------LLGSRSRLQDVPKIAQLIE 307
C.g.            MTFTFLDDYRLDFSIRS------------------------LMGSRSRLQDVPKIAQLVE 336
S.m.            MIFTFLDDYRLDFSIRS------------------------LLGSRSRLQDVPKIAQLVE 308
M.o.            MVFNFLDDYRLDFSIRS------------------------LVGSRSRLQDVPKIAQLVE 313
P.a.            MTFTFLDDYRLDFSIRS------------------------LLGSRSRLQDVPKIAQLVE 315
F.o.            MIFNFLDDYRLDFEIRS------------------------LLGSRSKLHNVPKIAQLVE 311
V.d.            MTFNFLDDYRLDFSIGS------------------------LLGSRSKLEDVPKIAQLIE 308
A.n.            IAFSFLPDYRLDLSVRS------------------------LIGSRSRLQDVPKVAQLVE 354
T.t.            LAFSFLPDYRLDISVRS------------------------LIGSRSRLQDVPKVAQLVE 350
C.i.            LAFSFLPDYRLDLSVRS------------------------LIGSRSRLQDVPKVAQLVE 360
P.b.            LAFSFLPDYRLDLSVRS------------------------LIGSRSRLQDVPKVAQLVE 390
S.s.            LAFCFLDDYRLDLSIRS------------------------LVGSRSRLQDVPKIAQLIE 300
B.f.            LAFCFLDDYRLDLSIRS------------------------LVGSRSRLQDVPKIAQLIE 305
P.t.            LAFTFLDDYRLDLSVRS------------------------LVGSRSRLQDVPKIAQLIE 341
S.c.            LMFSFSPEYRMEFEIKS------------------------LIGSRSKLENIPKIGSVIE 387
K.l.            LMFSFNPEYRMELEVKS------------------------LIGARSKLENIPKIASLIE 386
C.a.            LMFSFSPDYRLEFIVKS------------------------LIGSRAKLQDVPKISSLIE 373
S.p.            MNLSFDPNFVISLQVSS------------------------LVGARSKLQDIPKITQLIE 299
U.m.            LPTANPNPSSSSSSSATPPRSRHQLHFSLHPDFALEASATSLLGSRAKLQDIPKIEQLLI 369
C.n.            IHVNLLPDFHLNLKVTS------------------------LLGSRAKLQDIPKLEQLIV 256
R.o.            KP------------------------------------------------KIPLERYIAI 191
B.d.            QQPLYKSQASETSTS-----------------------------------DLGHHTYVSV 198
                                                                  .:     :

N.c.            SRLHRWFDERCVEPRFQEIALPNMWPRKKNTRG---GDETISDVER-------------S 352
G.z.            SRLHRWFDERAVEPRFQEIALPSLWPRKKNTRG---PDDGLAEGSV-------------P 351
C.g.            SRLHRWFDERCVEPRFQEIALPSMWPRKKNTRG---GDEAIADAER-------------S 380
S.m.            SRLHRWFDERCVEPRFQEIALPNMWPRKKNTRG---GDETISDVER-------------S 352
M.o.            SRLHRWFDERCVEPRFQEIPLPSMWPRKKNARG---GDDAIADVER-------------S 357
P.a.            SRLHRWFDERCVEPRFQEIELPSMWPRKKNTRG---GDEIIANIEQ-------------S 359
F.o.            ARLHRWFDERAVEPRFQEIALPSLWPRKKNTRG---PEDASTERSE-------------S 355
V.d.            ARLHRWFDERCVEPRFQEIALPSLWPRKKNTRG---PDDGMAEGNIG------------S 353
A.n.            ARVHAWFEERVVEPRVQVVGLPDLWPRMGRTGVRT-GDESETGSNTASRPAMSVDMS--S 411
T.t.            ARVQSWFEDRVVEPRVQLVALPGIWPRMGRTGVRA-QEDHDAVSDSDDP-EAKAAKSGFT 408
C.i.            ARVQAWFEERVVEPRVQVVALPGIWPRMGRTGVRG-QEEQQEVGSSGNAGVSTANVSMLG 419
```

```
P.b.          ARVQSWFEERVVEPRVQVVGLPNIWPRMGRTGLRSSQEEPEAGSGSVEIPVMTSPGADGV 450
S.s.          ARLHTWFDERCVEPRFQQIELPSLWPRKKNTRG---GEDLDTGSEAG------------G 345
B.f.          ARLHTWFDERCVEPRFQQIELPSLWPRKKNTRG---GEDLDTGSDAG------------G 350
P.t.          SRVHAWFDERAVEPRFQQIVLPSLWPRKHNTRGGA-PEDTEAAVEGEEGLDEDDFAVVDG 400
S.c.          YQIKKWFVERCVEPRFQFVRLPSMWPRSKNTRE---EKPTEL------------------ 426
K.l.          YQISKWFVERCVEPRFQFVKLPSMWPRSKNTRK---EKTDTDDSVS-------------- 429
C.a.          NQLRTWFIERCVEPRFQVVRLPSLWPRTKNTREPVTKKTTTTPSTT-------------- 419
S.p.          SRIRQWFTNRCVSPQFQQIAIPNLWPTSAKEGHARSHAPQEESSNED------------- 346
U.m.          SRLRGWIMDRFVWPRYWSLTLPNLVPSPAASRSFSAAAAANRHTDAASVGSSGGHGQINV 429
C.n.          SRLRNLVQDRFVHPNHISLALPRILSPSVSSTPILEGLGEGAVDAMKDAVSDGMKRMVED 316
R.o.          SSYSDFILD-------LQIKVPNMWK---------------------------------- 210
B.d.          SVLDDFLLD---------FDVRSLLG---------------------------------- 215
                     . :         . :  :

N.c.          MSKAKG----------VDIAKDVREEARKEIEAEAHGGADRV---------------PDS 387
G.z.          IGRSKG----------RDASRDPQ-EEARG---DAEIRDR-----------------GT 379
C.g.          MGKSKG----------MEMAKEMRTEARREVEAETDVRADRG---------------HES 415
S.m.          MNKTKG----------VDIAKDVREEARKEIEAEAHGGADRV---------------PDS 387
M.o.          MSKAKG----------ADVARDLR----REVLVEAEARQAAQ---------------RDS 388
P.a.          INKAHG----------GAIAKEAR----QELDTETDG---------------------- 382
F.o.          IGRSRG----------NDAGRDHR-EEIRGRGGDAEIRDRGA---------------GGT 389
V.d.          VGRSKG----------RDVGQDIR-AEARK-EVEAEASSRTD---------------RAQ 386
A.n.          PGHLQGD--------GGNHEEELRFRGLGPRPPLPFDAVSRTSSYQVE---------TGA 454
T.t.          PVNANRD--------GPQTPRDLNMDGLRYRRGNGGGQSTPDNYENLP---------QGD 451
C.i.          ARDAGAE--------GSHATRDADMEGLRYRRNASPGDETSGVRYSPQ---------NQD 462
P.b.          SGGGGSG--------GGSGGGGGGMRGIDRGLSGREAGYEALRYRHAA---------CGG 493
S.s.          IGRARS----------RDVERDLREEARKEVEAETGVRVGRSKLGVSLDVPDVGLD-GGS 394
B.f.          IGRARS----------RDVERDLREEARKEVEAETGIRVGRSKLGVSLDVPDE-----GS 395
P.t.          NGTAPGSTSYIPTPIAENATLEERIEAEGAKMREAEIRAGVRKPSASQERSRGRDDRADG 460
S.c.          ------------------------------------------------------------
K.l.          ----------------VKSND--------------------------------------- 434
C.a.          ----------------VNGTSAATVTTPGEYVNSNI------------------------ 439
S.p.          ------------------------------------------------------------
U.m.          GTGVERTDASVIQPGHHESARQEMLHAASERPS------LASSRPPHVRSSSSGLRANGM 483
C.n.          FMGENPVEGALNGQGEEQWLDDDFPPTPLVQPPGTFPTLSVSSRQSHRQSLPPSRPQSTT 376
R.o.          -----------------EQEEQEE------------------------------------ 217
B.d.          ----------------HRTKVKDLPKLTSLISN------SLRSVFISEMVFPACKMIKIP 253

N.c.          LRYRHRPRADEEFPGAGSMPGSMPG-SMP------------------------------- 415
G.z.          IRQRRGTRGSDSDD--FSMPGSLPSFQIPTP----------------------------- 408
C.g.          LRYRRRPQTDDSFSTSGSMPGSMPDLDIPT------------------------------ 445
S.m.          LRYRHRPRAEEEFPGAGSMPGSMPG-SMP------------------------------- 415
M.o.          LRYRR-PRADDAFP----MPGSLAVDD--------------------------------- 410
P.a.          LRYRRRPVGDDTYSVSGSMPGSLPGIDMPT------------------------------ 412
F.o.          VRHRRGTRSNDSDD--FSMPGSLPDFQVAAS----------------------------- 418
V.d.          ESLRR-RRLRSEDE--LIMPGSLPDFVQ-------------------------------- 411
A.n.          PRSPSLTRERSLG-DDFHMPGSMPEAPGAQ------------------------------ 483
T.t.          GHPQPTSG------EQFRIPGSLPGTSAIA------------------------------ 475
C.i.          SREQACRD------DPFRIPGSLPDVVPVT------------------------------ 486
P.b.          HQNQSGRDGGRGGNEQFAMPGSMPDTVTET------------------------------ 523
S.s.          EEGLRFRRRSRGRGDEYAMPGSMPGLSMA------------------------------- 423
B.f.          EDGLRFRRKSKGR-DEYAMPGSMPGLSMT------------------------------- 423
P.t.          MRWRGEQRERTTGPGRPKLQSRTTTGIVKAIPGALPR----------------------- 497
S.c.          ------------------------------------------------------------
K.l.          ------------------------------------------------------------
C.a.          ------------------------------------------------------------
S.p.          ------------------------------------------------------------
U.m.          GSVEAWRAHAAGVYNQPSHQHATSLNQAALLRAHVGGGGATDCAPGSDVNIEVPGSYRGS 543
C.n.          QGQPQLFYRRPLIHPTQSYPHYNTYTLDPQIPHSVSYRHPPRGSHVHNPPETPVPQRPSH 436
R.o.          ------------------------------------------------------------
B.d.          NGEELFGDIANAVDENSHDVFLNQENGSETLKSA-------------------------- 287
```

```
N.c.            ---------------------------------------
G.z.            ---------------------------------------
C.g.            ---------------------------------------
S.m.            ---------------------------------------
M.o.            ---------------------------------------
P.a.            ---------------------------------------
F.o.            ---------------------------------------
V.d.            ---------------------------------------
A.n.            ---------------------------------------
T.t.            ---------------------------------------
C.i.            ---------------------------------------
P.b.            ---------------------------------------
S.s.            ---------------------------------------
B.f.            ---------------------------------------
P.t.            ---------------------------------------
S.c.            ---------------------------------------
K.l.            ---------------------------------------
C.a.            ---------------------------------------
S.p.            ---------------------------------------
U.m.            VAGAGIELASSSNASSVLPTGAHHSSGLRNRPGFVQ--- 579
C.n.            GQGRMSTTSSLTPSQSQSQFRFRGQFASGVTPGQVGTSR 475
R.o.            ---------------------------------------
B.d.            ---------------------------------------
```
