# Supplementary material for: Analysis of Mutations in Neurospora crassa ERMES Components Reveals Specific Functions Related to β-Barrel Protein Assembly and Maintenance of Mitochondrial Morphology
Source: PLoS One. 2013 Aug 5;8(8):e71837. doi: 10.1371/journal.pone.0071837 (PMC3733929; doi:10.1371/journal.pone.0071837)
Supplement: Figure S4 — Cysteine mutants have normal TOM complex and Tim8/13 levels. (PDF) [file pone.0071837.s004.pdf]

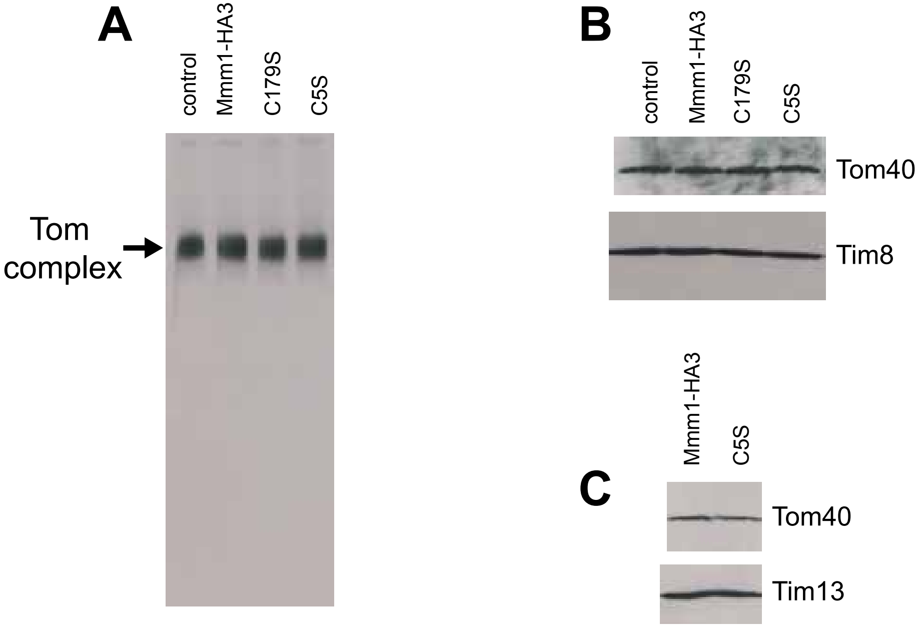

SUPPORTING INFORMATION FIGURE S4. Cysteine mutants have normal TOM complex and Tim8/13 levels. A. Mitochondria were isolated from the indicated strains. Aliquots containing 20 µg of mitochondrial protein were dissolved in 1% digitonin and subjected to BNGE. Proteins in the gel were electroblotted to PVDF membrane and immunostained with antibody to Tom40. The position of theTOM complex is indicated. (Control is wild type strain NCN251). B. Mitochondria were isolated from the indicated strains and 30 µg of mitochondrial protein was subjected to SDS-PAGE. Proteins in the gel were electroblotted to nitrocellulose and immunostained with the antibodies indicated on the right. C. As for panel B.
