# Supplementary material for: Analysis of Mutations in Neurospora crassa ERMES Components Reveals Specific Functions Related to β-Barrel Protein Assembly and Maintenance of Mitochondrial Morphology
Source: PLoS One. 2013 Aug 5;8(8):e71837. doi: 10.1371/journal.pone.0071837 (PMC3733929; doi:10.1371/journal.pone.0071837)
Supplement: Figure S5 — Alkali extraction of mitochondria from Mmm1 mutant A116-124. (PDF) [file pone.0071837.s005.pdf]

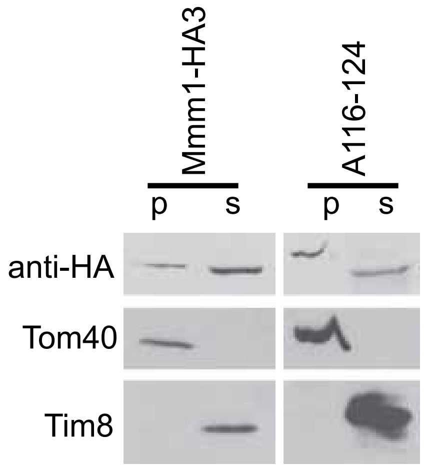

SUPPORTING INFORMATION FIGURE S5. Alkali extraction of mitochondria from Mmm1 mutant A116-124. Crude mitochondria (25 µg for Mmm1-HA3 or 200 µg for mutant A116-124) isolated from the indicated strains were treated with 0.1 M sodium carbonate at pH 11.0. Samples were then ultracentrifuged to pellet the membrane sheets. Proteins in the supernatant were precipitated with trichloroacetic acid. Pellet (pel) and supernatant (sup) fractions were then subjected to SDS-PAGE, transferred to nitrocellulose and analyzed by Western blot using antibodies to the indicated proteins.
